# Celastrol mitigates staphyloxanthin biosynthesis and biofilm formation in *Staphylococcus aureus* via targeting key regulators of virulence; in vitro and in vivo approach

**DOI:** 10.1186/s12866-022-02515-z

**Published:** 2022-04-15

**Authors:** Fatma Al-zahraa A. Yehia, Nehal Yousef, Momen Askoura

**Affiliations:** Department of Microbiology and Immunology, Faculty of Pharmacy, Zagazig University, Zagazig, 44519 Egypt

**Keywords:** *Staphylococcus aureus*, Celastrol, Virulence, Staphyloxanthin, Biofilm

## Abstract

**Background:**

*Staphylococcus aureus* is a leading cause of human infections. The spread of antibiotic-resistant staphylococci has driven the search for novel strategies to supersede antibiotics use. Thus, targeting bacterial virulence rather than viability could be a possible alternative.

**Results:**

The influence of celastrol on staphyloxanthin (STX) biosynthesis, biofilm formation, antibiotic susceptibility and host pathogenesis in *S. aureus* has been investigated. Celastrol efficiently reduced STX biosynthesis in *S. aureus*. Liquid chromatography-mass spectrometry (LC–MS) and molecular docking revealed that celastrol inhibits STX biosynthesis through its effect on CrtM. Quantitative measurement of STX intermediates showed a significant pigment inhibition via interference of celastrol with CrtM and accumulation of its substrate, farnesyl diphosphate. Importantly, celastrol-treated *S. aureus* was more sensitive to environmental stresses and human blood killing than untreated bacteria. Similarly, inhibition of STX upon celastrol treatment rendered *S. aureus* more susceptible to membrane targeting antibiotics. In addition to its anti-pigment capability, celastrol exhibits significant anti-biofilm activity against *S. aureus* as indicated by crystal violet assay and microscopy. Celastrol-treated cells showed deficient exopolysaccharide production and cell hydrophobicity. Moreover, celastrol markedly synergized the action of conventional antibiotics against *S. aureus* and reduced bacterial pathogenesis in vivo using mice infection model. These findings were further validated using qRT-PCR, demonstrating that celastrol could alter the expression of STX biosynthesis genes as well as biofilm formation related genes and bacterial virulence.

**Conclusions:**

Celastrol is a novel anti-virulent agent against *S. aureus* suggesting, a prospective therapeutic role for celastrol as a multi-targeted anti-pathogenic agent.

**Supplementary Information:**

The online version contains supplementary material available at 10.1186/s12866-022-02515-z.

## Background

*Staphylococcus aureus* is an aggressive pathogen causing a diverse range of human infections. It has the ability to thrive in a wide range of host niches, from skin to abiotic devices and deep-seated tissues, which renders it difficult to eliminate, causing recurrent infections [1]. The main concerning issue regarding this pathogen is the overwhelming rise of resistant strains to various antibiotics [2]. This developed antimicrobial resistance renders treatment of *S. aureus* infections a serious concern, particularly in healthcare facilities [3]. Therefore, innovative anti-infective agents that work by blocking bacterial virulence factors should be employed rather than using conventional antibiotics to treat *S. aureus* infection [4]. A potential benefit of this approach is that there is no “life or death” selective pressure on bacteria, so it is less likely to evolve resistance [5].

*S. aureus* produces a multitude of virulence factors that help it infect the host and evadethe immune system efficiently [6]. One such virulence factor is the carotenoid staphyloxanthin (STX), which gives *S. aureus* a golden-yellow color appearance. STX is a C_30_ carotenoid membrane bound pigment that reacts with and deactivates the reactive oxygen species (ROSs) exerted by macrophages and host neutrophils, which enhances *S. aureus* resistance to immune clearance [7]. In addition, STX significantly regulates bacterial membrane mechanical properties, which is important for increasing *S. aureus* fitness and virulence potential [7]. The STX biosynthesis is mediated by the *crtOPQMN* operon, which encodes five distinct enzymes in addition to aldehyde dehydrogenase enzyme *aldH*. The biosynthesis of STX begins with the condensation of two molecules of farnesyl diphosphate to form 4,4`-diapophytoene, which is catalyzed by the dehydrosqualene synthase CrtM. Then 4,4`-diapophytoene undergoes dehydrogenation by dehydrosqualene desaturase CrtN to give 4,4′-diaponeurosporene that is finally oxidized, glycosylated, and esterified to give STX [8].

Another fundamental characteristic of *S. aureus* is its capability to develop biofilm on biotic as well as abiotic surfaces, which has a significant role in bacterial resistance to various antimicrobials and persistence within the host [9]. Biofilm is an extracellular complex polymeric structure that surrounds and protects bacterial cells [10]. The polysaccharide intracellular adhesin (PIA), which is encoded by the *ica* operon, is the major component in staphylococcal biofilms. PIA is crucial in biofilm as it promotes bacterial adherence and biofilm structure. Moreover, extracellular DNA and various surface proteins were found to markedly contribute to biofilm stability [11]. It has been shown that bacteria within biofilm exhibit a higher degree of resistance to antimicrobials. Several mechanisms, including poor antibiotic penetration, genetic manipulation, degrading matrix enzymes, a high degree of gene transfer, and persister cell development, could account for this bacterial resistance to antimicrobials [12]. Therefore, inhibition of bacterial biofilm formation has been recently regarded as one of the successful therapeutic strategies to counteract persistent bacterial infections [13].

Redox-active phytochemicals have recently attracted interest owing to the diversity of their biological activity [14]. Celastrol is a redox-active quinone methide compound, which is isolated from the roots of the thunder god vine, *Tripterygium wilfordii*. Celastrol has a wide range of therapeutic uses, such as anti-inflammatory, hepatoprotective, neuroprotective, and immunomodulatory effects [15, 16]. Additionally, celastrol has been reported as a promising treatment for obesity via increasing leptin sensitivity and augmentation of energy expenditure [17]. In addition, celastrol improves lipid metabolism and mitigates high fat-mediated oxidative injury by reducing levels of total cholesterol, triacylglycerol and low-density lipoprotein cholesterol [18]. Importantly, the pharmacokinetic properties of celastrol have been precisely investigated in previous studies [19, 20]. Unfortunately, there have been very few studies that have characterized the anti-biofilm and anti-virulence activity of celastrol against various pathogens, notably bacterial pathogens. For instance, celastrol has been reported as a non-competitive inhibitor of fungal squalene synthase [21]. However, neither the anti-pigment nor the anti-biofilm potential of celastrol against *S. aureus* has been characterized yet. Owing to the structural similarity between fungal squalene synthase and *S. aureus* dehydrosqualene synthase (CrtM), celastrol could be a potent inhibitor of the carotenoid STX biosynthesis in *S. aureus*. The present study aims to characterize the anti-pigment and anti-biofilm activity of celastrol against *S. aureus.* The results of the current study could be helpful in the use of celastrol in treatment of *S. aureus* infections.

## Materials and methods

### Bacterial strains and growth conditions

*S. aureus* ATCC 6538 (GenBank accession number CP020020) and one hundred *S. aureus* clinical isolates recovered from patients at the University Hospital, Zagazig, Egypt, were involved in the current study. Clinical isolates were obtained from patients suffering from skin and soft tissue infections (49 isolates), respiratory tract infections (43 isolates), and urinary tract infections (8 isolates). Bacterial isolates were identified according to Washington et al. [22], further confirmed by using API Staph (Biomérieux, France) and a matrix-assisted laser desorption/ionization-time-of-flight mass spectrometer (MALDI-TOF/MS) using the VITEK MS system (Biomérieux, Inc., Durham, USA) and categorized according to pigment production. Those isolates showing the highest and lowest pigment content were selected for further work as representatives for pigmented and non-pigmented isolates, respectively. Bacterial cultures were grown aerobically in trypticase soy broth (TSB), and TSB with 1% glucose (TSBG) was used for biofilm and virulence assays.

### Carotenoid pigment extraction and estimation

Carotenoid pigment extraction was done following Kossakowska-Zwierucho et al. [23]. Overnight bacterial cultures with an optical density (OD_600_) of 2.0 (4 × 10^9^ colony forming units (CFUs) in 10 mL volume) were harvested at 4000 rpm for 10 min followed by washing with phosphate buffer saline (PBS). Bacterial cells were added to 1 mL of 99% methanol and agitated for 30 min at 55 °C in the dark until the pellet became colorless. After centrifugation at 8000 rpm for 15 min at 4 °C, the absorbance of the supernatant was measured spectrophotometrically at 465 nm using a microplate reader (BIO Tek Synergy HT).

### Determination of minimum inhibitory concentration (MIC)

The MIC of celastrol (MCE; MedChemExpress, USA) was determined for *S. aureus* ATCC 6538 [24]. Serial twofold dilutions of celastrol were prepared in Mueller Hinton broth (MHB) in a microtiter plate. Bacterial suspensions (10^6^ CFU/mL) were added to each well containing celastrol. After incubation, MICs were determined in triplicate.

### Pigment inhibition assay

The influence of celastrol on pigment production was characterized. *S. aureus* ATCC 6538 and four clinical isolates with the highest pigment content were cultured in TSB in the presence of various concentrations of celastrol (280, 330, 360, 420 and 440 nM). Bacterial pigment was extracted and measured as described above. The half maximal inhibitory concentration values (IC_50_) were calculated by plotting the OD_465_ data to a normal dose-inhibition curve [25].

### Spectrometric quantification of intermediates of STX biosynthesis

Methanol extraction of carotenoid pigment for control untreated and celastrol treated cells was carried out as mentioned before. The methanolic extract containing various STX intermediates was measured spectrophotometrically at different wavelengths using a Synergy HT plate reader. The absorbance wavelengths used for quantification of 4,4′-diapophytoene, 4,4′-diaponeurosporene, 4,4′-diaponeurosporenic acid, and STX were 286 nm, 435 nm, 455 nm, and 465 nm, respectively [26].

### Liquid chromatography-mass spectrometry (LC-MS) analysis

The carotenoid pigment was collected from control untreated and celastrol treated cells as mentioned above and mixed with ethyl acetate and NaCl (2.5 M). Pigment dissolved in organic layer was separated, washed, and dried using the Zipvap nitrogen evaporator (Glas-Col, Terre Haute, IN, USA). A 10-μL aliquot was applied to the ACQUITY UPLC - BEH C_18_ Column (1.7 μm - 2.1 × 50 mm) and eluted under isocratic conditions with a solvent system consisting of water with acetonitrile with formic acid (0.1%) and formic acid (0.1%) at a flow rate of 0.2 mL/min. Carotenoids were structurally characterized using both HPLC retention time and mass fragmentation spectra by ESI-MS in negative ion acquisition mode using a XEVO TQD triple quadruple instrument [27].

### Molecular docking

The structural complexes of celastrol with dehydosqualene synthase (CrtM) were determined using in silico docking approach. Celastrol structure was obtained from PubChem (CID: 122724) and the 3D-crystal structure of dehydroxysqualene synthase CrtM (ID: 2ZCO) was recovered from the protein databank (PDB). In silico molecular docking was performed using AutoDockTools version 1.5.7 [28]. The predicted binding affinity of celastrol with CrtM is shown in kcal/mole and the best binding mode was selected and represented for analysis.

### Oxidative stress survival assay

The effect of celastrol on *S. aureus* survival to oxidative stress by hydrogen peroxide was determined as described by liu et al. [7]. *S. aureus* ATCC 6538 and the non-pigmented bacteria were incubated overnight in TSB with or without celastrol (440 nM). Cells were collected by centrifugation, washed, and adjusted to a concentration of 2.5 × 10^6^ CFU/ 250 μL. Hydrogen peroxide was added and the samples were incubated for 1 h. Bacterial survival was evaluated every 15 min by serial dilutions and plating on tryptone soya agar (TSA) plates for counting the surviving CFUs.

### Acid stress survival assay

*S. aureus* ATCC 6538 and the non-pigmented isolate were cultured in TSB with or without celastrol (440 nM). After 24 h, bacteria were harvested by centrifugation at 8000 rpm, washed, and diluted to 1× 10^6^ CFU/mL. Bacterial suspension was exposed to acid stress at pH 4 adjusted by acetic acid and incubated for 15 min. Bacterial survival was evaluated every 3 min by serial dilutions and plating on TSA plates for counting the surviving CFUs.

### Whole blood killing assay

Overnight cultures of *S. aureus* ATCC 6538 and the non-pigmented isolate with and without celastrol (440 nM) were adjusted to 6 × 10^8^ CFU/mL. Freshly drawn human blood (heparinized) was added to bacterial culture (3:1) and incubated for 2 h with shaking at 160 rpm. Bacterial viability was evaluated every 30 min by plating on TSA plates [7].

### Fourier transform infrared (FTIR) spectroscopy analysis

Changes in *S. aureus* membrane upon celastrol treatment were characterized using FTIR spectroscopy analysis. *S. aureus* ATCC 6538 cultures on TSA with celastrol (440 nM) were suspended in PBS to a concentration of 9 × 10^8^ CFU/mL. Bacterial cell suspension was centrifuged for 5 min at 4000 rpm and the cell pellet was subjected to FTIR analysis (Bruker Alpha FTIR). The spectrum was scanned between 4000 and 500 cm^− 1^ and spectra were plotted as transmittance versus wave number [29].

### Polymyxin B susceptibility test

Polymyxin diffusion assay was performed according to Valliammai et al. [30] with minor modification. *S. aureus* ATCC 6538 cultures were swabbed on TSA plates without and with celastrol (360, 420 and 440 nM). Wells were cut into the center of agar medium, and 20 μL of 1 mM polymyxin B were added to each well. After incubation, bacterial culture inhibition zones were measured in mm and plates were photographed.

### Biofilm inhibition assay

Biofilm inhibition quantitative assay was carried as described Stepanovic et al. [31]. Overnight culture of *S. aureus* ATCC 6538 in TSB was adjusted to 10^6^ CFU/mL in fresh TSBG. Bacterial suspensions were transferred to both polypropylene tubes and polystyrene microtiter plates with and without celastrol (360, 420 and 440 nM). The planktonic cells were discarded after 48 h and plates were washed. Plates were air-dried, and 99% methanol was added for 20 min as a fixing agent. The fixed biofilm was then stained with 1% crystal violet (CV) solution for 15 min and glacial acetic acid (33%) was finally added to solubilize CV. The absorbance was measured at 570 nm spectrophotometrically. The percentage of biofilm inhibition by celastrol was estimated as shown: % of inhibition = {(Control OD_570 nm_ − Treated OD_570 nm_)/Control OD_570 nm_}z × 100. Where the control refers to bacteria only and treated refers to bacteria treated with celastrol.

### Microscopical characterization of biofilm

The anti-biofilm activity of celastrol against *S. aureus* ATCC 6538 was characterized using both a light and a scanning electron microscope [32]. Biofilms were developed on polystyrene discs in the absence and presence of celastrol (360, 420 and 440 nM) as described before [31] and fixed with 2.5% glutaraldehyde. Fixed discs were subsequently washed again by PBS and dehydrated through serially graded ethanol solutions. Discs were dried and gold coated before imaging and examined using a JEOL scanning microscope (JSM - T100, Japan).

### Quantitative real-time PCR analysis

The effect of celastrol on the expression of *S. aureus* genes using qRT-PCR was assessed. These genes include genes responsible for STX biosynthesis (*crtM* and *crtN*), biofilm formation (*icaA*, *icaR*, *sarA* and agrA), oxidative stress response (*katA*, *sodA* and *sodM*), transcription regulation (*msaB*, *sigB*) and stress adaptation (y*jbH*). Briefly, total RNA was extracted from an overnight culture of both celastrol treated and untreated *S. aureus* using the TRIzol reagent following the manufacturer’s instructions. Extracted RNA was purified using a Qiagen RNeasy minikit and reverse-transcribed into cDNA using a QuantiTect-Reverse Transcription Kit (Qiagen, USA) and random primer hexamet. Amplification of cDNA was performed following the manufacturer’s protocol of Maxima SYBR Green/Fluorescein qPCR Master Mix. The expression level of tested genes was normalized to 16S rRNA. The primers used for qRT-PCR are shown in Table 1 [33–39]. Relative gene expression was calculated by the 2^-∆∆*CT*^ method [40].

**Table 1:** Primers used for qRT-PCR analysis

Gene name		Primer sequence (5`-3`)
***crtM*** **(F)**		GGTGTTGCTGGTACAGTAGGTGAAG
***crtM*** **(F)**		GCAACGATTCACCAAGTCTTCTTGCG
***crtN*** **(F)**		CAGTGATTGGTGCAGGTGTC
***crtN*** **(R)**		CATACGCCCGCCTACATTAT
***katA*** **(F)**		AAAGGTTCTGGTGCATTTGG
***katA*** **(R)**		AACGCAAATCCTCGAATGTC
***sodA*** **(F)**		TGC ACGCTTTGGTTCAGGTTGGG
***sodA*** **(R)**		GCGCCAATGTAGTCAGGGCGTTTG
***sodM*** **(F)**		CCGGAAGCGATGAGGATGTCAGTC
***sodM*** **(R)**		TGCCCCACTGCGCTTTGATGT
***icaA*** **(F)**		CTGGCGCAGTCAATACTATTTCGGGTGTCT
***icaA*** **(R)**		GACCTCCCAATGTTTCTGGAACCAACATCC
***icaR*** **(F)**		TGCTTTCAAATACCAACTTTCAAGA
***icaR*** **(R)**		ACGTTCAATTATCTAATACGCCTG
***sarA*** **(F)**		CAAACAACCACAAGTTGTTAAAGC
***sarA*** **(R)**		TGTTTGCTTCAGTGATTCGTTT
***agrA*** **(F)**		TGATAATCCTTATGAGGTGCTT
***agrA*** **(R)**		CACTGTGACTCGTAACGAAAA
***sigB*** **(F)**		CGTCTCGGAACATGTACACTCCAAG
***sigB*** **(R)**		GTCCTTTGAACGGAAGTTTGAAGCC
***cspA*** **(*msaB*) (F)**		TTTATCGAAGTTGAAGGAGAAAATG
***cspA*** **(*msaB*) (R)**		ACTCAACAGCTTGACCTTCTTCTAA
***yjbH*** **(F)**		AAGCCCCTTCTCTCGTTTTC
***yjbH*** **(R)**		TTTAAAAGTTTTTCTGGCCATTC
**16 s** ***rRNA*** **(F)**		ACTCCTACGGGAGGCAGCAG
**16 s** ***rRNA*** **(R)**		ATTACCGCGGCTGCTGG

*F* Forward, *R* Reverse

### Checkerboard microdilution assay

Checkerboard assay was performed as previously described [41] to measure synergy between celastrol and different antibiotics against *S. aureus* ATCC 6538. The tested antibiotics include ampicillin (AMP), cefotaxime (CTX), ciprofloxacin (CIP), azithromycin (AZM), and gentamycin (CN). The fractional inhibitory concentration index (FICI) using fractional inhibitory concentration (FIC) was calculated using the following formula: FIC of celastrol = MIC celastrol in combination/MIC of celastrol alone; FIC of antibiotic = MIC of antibiotic in combination/MIC of antibiotic alone; FIC index (FICI) = FIC of celastrol + FIC of antibiotic. “Synergy” was defined when FICI was ≤0.5; while “additive” means 0.5 ≤ FICI ≤1.0; and “indifferent” when the FICI is between 1 and 4.

### In vivo murine infection assay

The role of STX in *S. aureus* pathogenesis was characterized using mice infection model [42]. *S. aureus* ATCC 6538 was incubated in the absence and presence of celastrol (440 nM). Cells were collected and resuspended in PBS to achieve a bacterial density of 2.5 × 10^7^ CFU/mL. Three-week-old healthy albino mice were intrabdominally injected with bacterial suspensions (100 μL). Mice were randomly divided into five groups, with six mice in each one. Mice in the first, second and third groups were injected with celastrol treated, untreated *S. aureus* ATCC 6538 and non-pigmented *S. aureus* isolate, respectively. As negative controls, a six-mouse group was injected with sterile PBS (100 L), as well as another group was left uninjected. Mice were sacrificed 24 h post infection, and organs were removed aseptically and weighed. Isolated organs were homogenized and serially diluted in PBS before being finally plated on MH agar plates for enumeration of CFUs. The bacterial burden in mouse organs was determined and expressed as CFU/g. In addition, fragments of isolated organs were fixed in buffered formalin (10%) for histopathological examination. The statistical analysis was performed using Mann–Whitney U analysis (*P* < 0.05 is considered significant).

### Statistical analysis

All experiments were performed in triplicate, and the results were represented as the mean ± standard error. Statistical analyses were accomplished with GraphPad Prism 5 software using Student *t*-tests or one-way ANOVA, unless otherwise mentioned.

## Results

### Quantitative estimation of carotenoid pigment production by *S. aureus* isolates

**Fig. 1:**
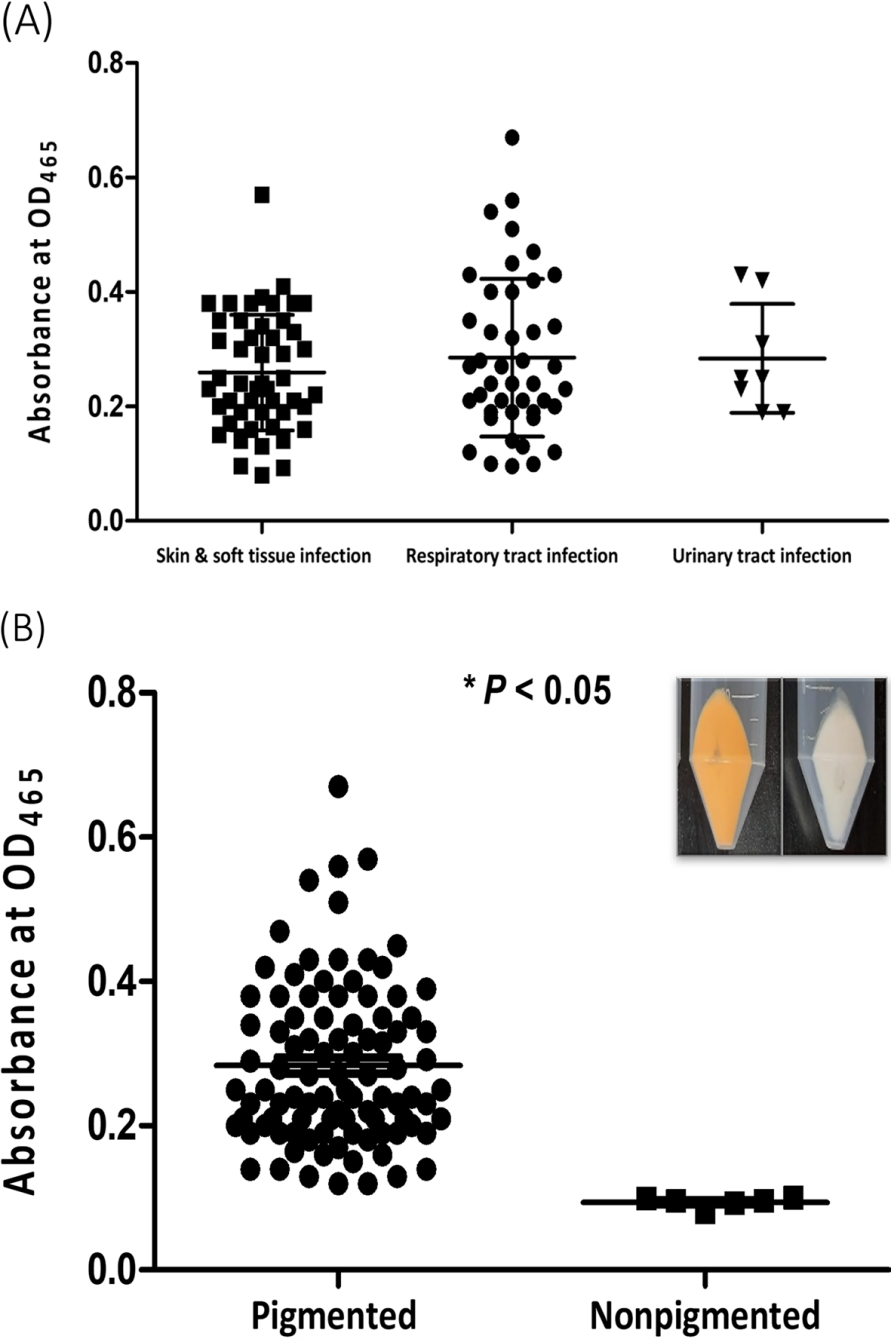
Quantitative estimation of carotenoid pigment production by *S. aureus* isolates. (**A**) Carotenoid quantification of *S. aureus* isolates from different clinical sources. (**B**) Grouping of *S. aureus* clinical isolates according to the absorbance (OD_465_) of extracted carotenoids. (Inset image represents pigmented and non-pigmented cell pellets). Each dot represents the mean OD value of each isolate from triplicate experiments

*S. aureus* clinical isolates were grouped according to their carotenoid content (Fig. 1A). There was no significant difference (*P* > 0.05) in carotenoid content between *S. aureus* isolates from different clinical sources. *S. aureus* clinical isolates that have a carotenoid absorbance (OD_465_) > 0.1 were considered pigmented isolates (94/100 isolates). Whilst those isolates that have carotenoid absorbance (OD_465_) ≤ 0.1 were considered non-pigmented isolates (6/100 isolates) (Fig. 1B).

### Determination of MIC of celastrol

The broth microdilution assay showed that MIC of celastrol against *S. aureus* ATCC 6538 is 1 μg/mL (2200 nM). The sub-MICs of celastrol (360, 420 and 440 nM) did not interfere with bacterial growth (Fig. S2A). Additionally, *S. aureus* metabolic activity was measured using the alamar blue assay, showing that celastrol treated cells are metabolically active similar to untreated cells (Fig. S2B). These findings clearly show that exposure of *S. aureus* to sub-MICs of celastrol does not affect either bacterial viability or metabolic activity.

### Celastrol decreases STX production

**Fig. 2:**
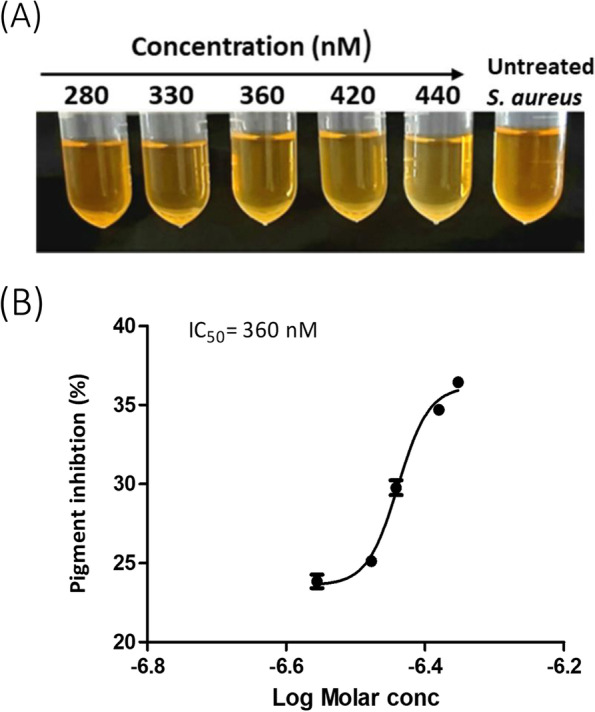
In vitro pigment inhibition by celastrol. (**A**) Inhibition of *S. aureus* ATCC 6538 pigment production in the presence of increasing concentrations of celastrol. (**B**) Half maximal inhibitory concentration (IC_50_) of celastrol against *S. aureus* ATCC 6538. Data shown represent the mean ± standard error from triplicate experiments

The influence of celastrol on STX production by *S. aureus* ATCC 6538 was examined. There was a concentration dependent reduction of the golden-yellow carotenoid pigment (Fig. 2A) with a half maximal inhibitory concentration (IC_50_) of 360 nM (Fig. 2B). Similarly, celastrol negatively affects STX production in pigmented *S. aureus* clinical isolates recovered from different sources (Fig. S3A-D). These findings demonstrate that celastrol has potent anti-staphyloxanthin activity on *S. aureus*, both the standard strain and those isolated from various clinical sources.

### Celastrol interacts with CrtM and leads to the accumulation of farnesyl diphosphate

**Fig. 3:**
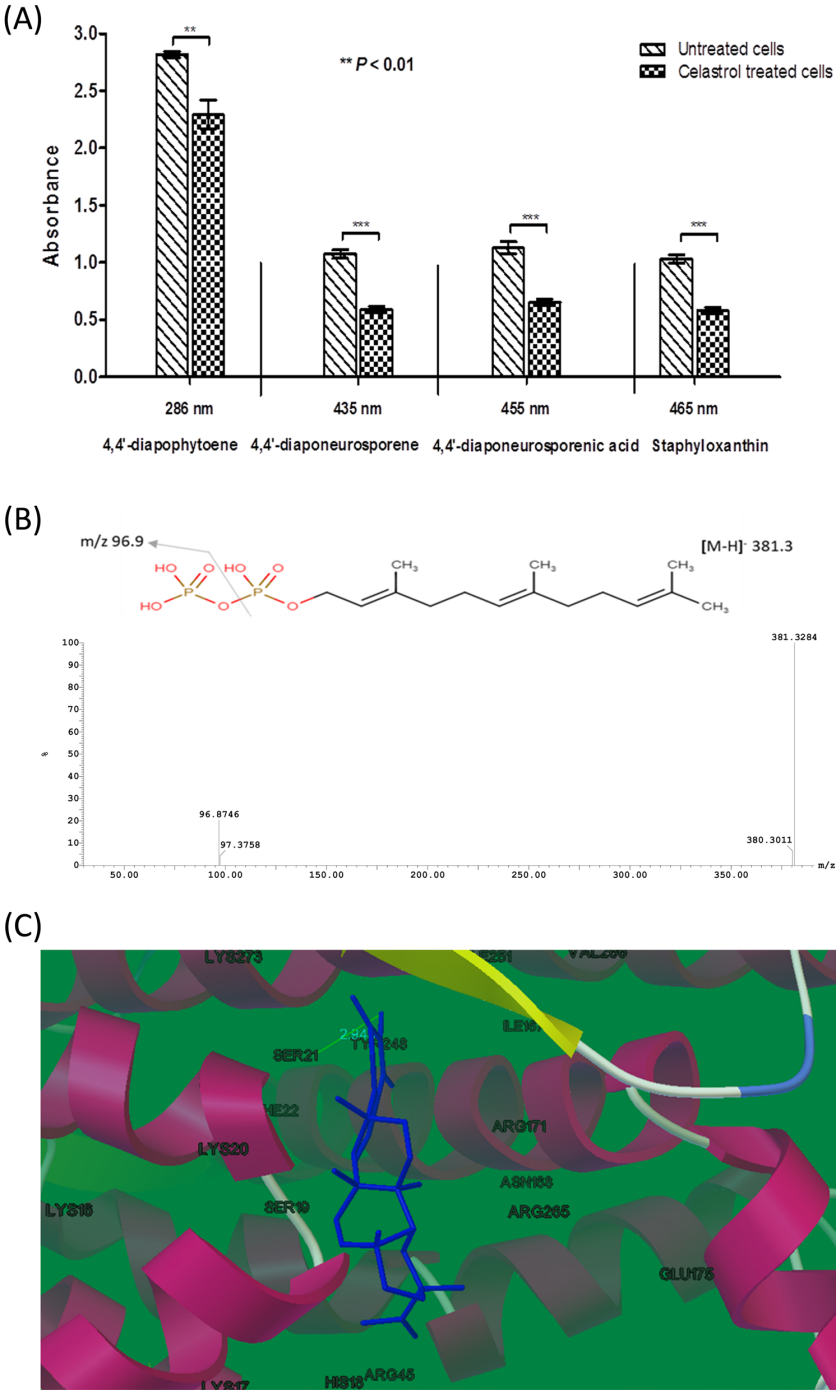
(**A**) Celastrol decreased production of STX as well as its intermediates (4–4’diapophytoene 4,4`-diaponeurosporene and 4–4` diaponeurosporenic acid). (**B**) LC-MS/MS of accumulated farnesyl diphosphate in celastrol treated *S. aureus*. (**C**) Molecular docking analysis of celastrol with CrtM by ribbon 3D-structure. Data shown represent the mean ± standard of error from triplicate experiments. A *P* value < 0.05 was considered statistically significant using Student’s *t* test

The target for celastrol inhibitory action on carotenoid production by *S. aureus* was characterized herein. The methanolic extracts of *S. aureus* carotenoids were subjected to both spectrometric quantification and LC-MS. Celastrol treatment revealed a significant reduction of STX as well as its intermediates, including 4,4`-diapophytoene, 4,4`-diaponeurosporene and 4,4`-diaponeurosporenic acid (Fig. 3A). This finding suggests that celastrol may interfere with the initial condensation of farnesyl diphosphate catalyzed by CrtM to form 4,4′-diapophytoene, thereby interfering with the synthesis of subsequent intermediates. In agreement, LC-MS analysis revealed an additional peak at 19.3 min with a molecular weight of 382.3 (Fig. 3B), which was identified as farnesyl diphosphate by LC-MS/MS. In support of these findings, molecular docking analysis elucidated the ability of celastrol to interact with *S. aureus* CrtM (Fig. 3C). Celastrol interacts with CrtM active sites with a binding energy of - 9.15 kcal/mol and exhibited one hydrogen-bonding interaction with Ser A: 21. These results clearly suggest that celastrol exerts its inhibitory effect on *S. aureus* by way of CrtM-mediated inhibition of STX production.

### Celastrol sensitizes *S. aureus* to both oxidative and acid stress as well as human blood killing

**Fig. 4:**
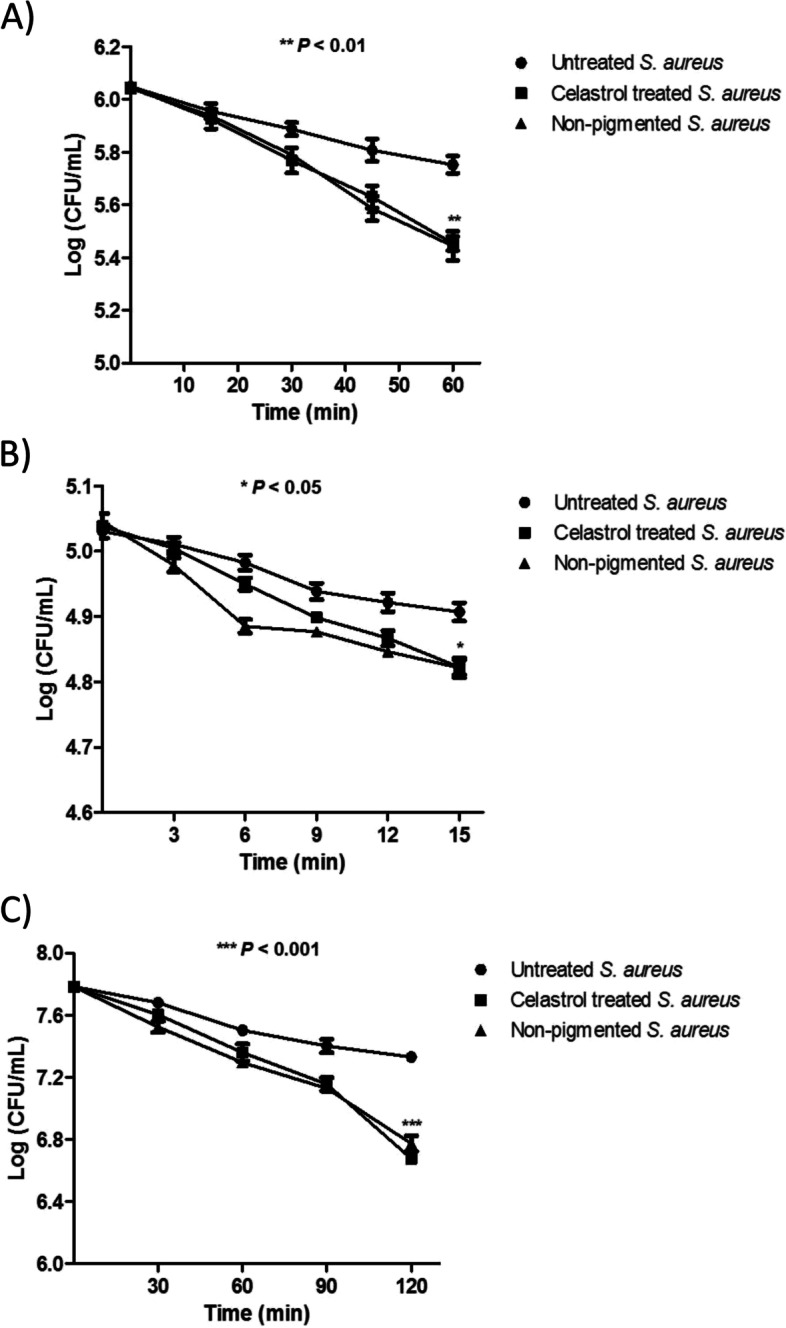
Reduced survival of celastrol treated *S. aureus* ATCC 6538 in presence of; (**A**) hydrogen peroxide, (**B**) acetic acid (pH 4) and (C) human blood cells, (•) untreated *S. aureus*, (■) celastrol treated *S. aureus* and (▲) non-pigmented *S. aureus* isolate. Data shown represent the mean ± standard of error from triplicate experiments. A *P* value < 0.05 was considered statistically significant using ANOVA test

The percentage of bacterial survival of celastrol treated *S. aureus* under oxidative and acid stress was determined and compared with that of untreated bacteria. Celastrol treatment significantly decreased (*P* < 0.05) *S. aureus* survival to oxidative stress (25% ± 3.1) and acidic stress (60% ± 0.8) when compared to control (50% ± 3.4 and 75% ± 0.7, respectively) (Fig. 4A-B). Furthermore, celastrol treated *S. aureus* was found to be more susceptible to immune clearance in human blood as compared with untreated bacterial cells. Celastrol treated *S. aureus* survival to whole blood killing was five times lower than that of untreated *S. aureus* (7.6% ± 0.4 vs. 34% ± 2.1; Fig. 4C).

### Celastrol affects bacterial membrane integrity and renders cells more susceptible to membrane targeting antibiotics

**Fig. 5:**
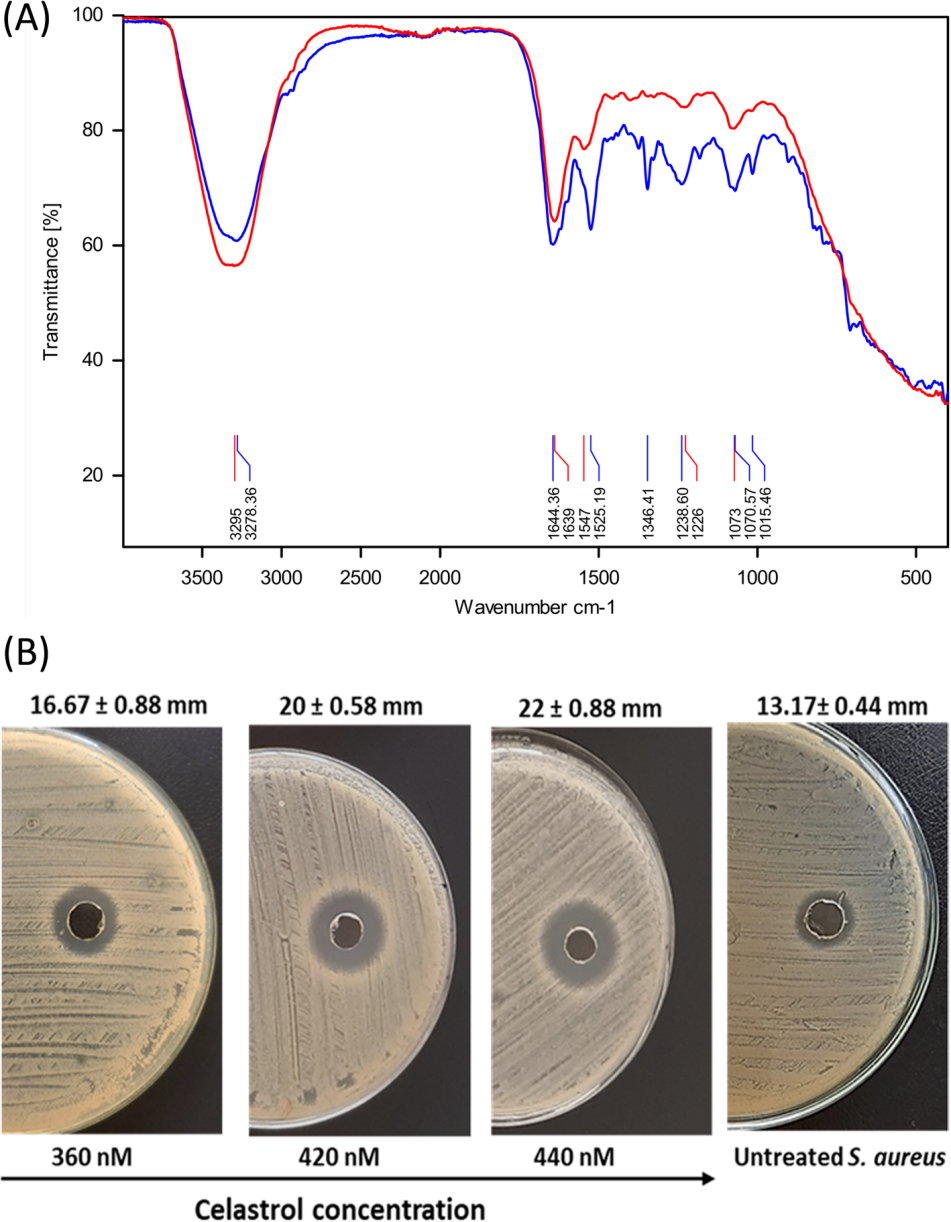
(**A**) The raw spectra of *S. aureus* ATCC 6538 cells and celastrol treated cells within wavenumber 4000–500 cm^− 1^. Blue and red lines represent control untreated and celastrol treated cells respectively. (**B**) Cup diffusion assay showing increased susceptibility of celastrol treated *S. aureus* to polymyxin B as compared with untreated cells. Inhibition zone diameter is represented as the mean ± standard error from triplicate experiments

FTIR spectroscopic analysis displayed marked changes in the spectral profile of celastrol treated *S. aureus* cells relative to untreated cells (Fig. 5A). The variation in bands (1500–1200 cm^− 1^) and (1200–900 cm^− 1^) corresponding to membrane phospholipids and polysaccharides, respectively, validated the effect of STX inhibition on *S. aureus* membrane. Additionally, celastrol treated cells showed increased susceptibility to polymyxin B in a dose-dependent manner as indicated by increased inhibition zones (Fig. 5B) which further confirms that celastrol affects the membrane integrity of *S. aureus.*

### Celastrol inhibits *S. aureus* biofilm formation

**Fig. 6:**
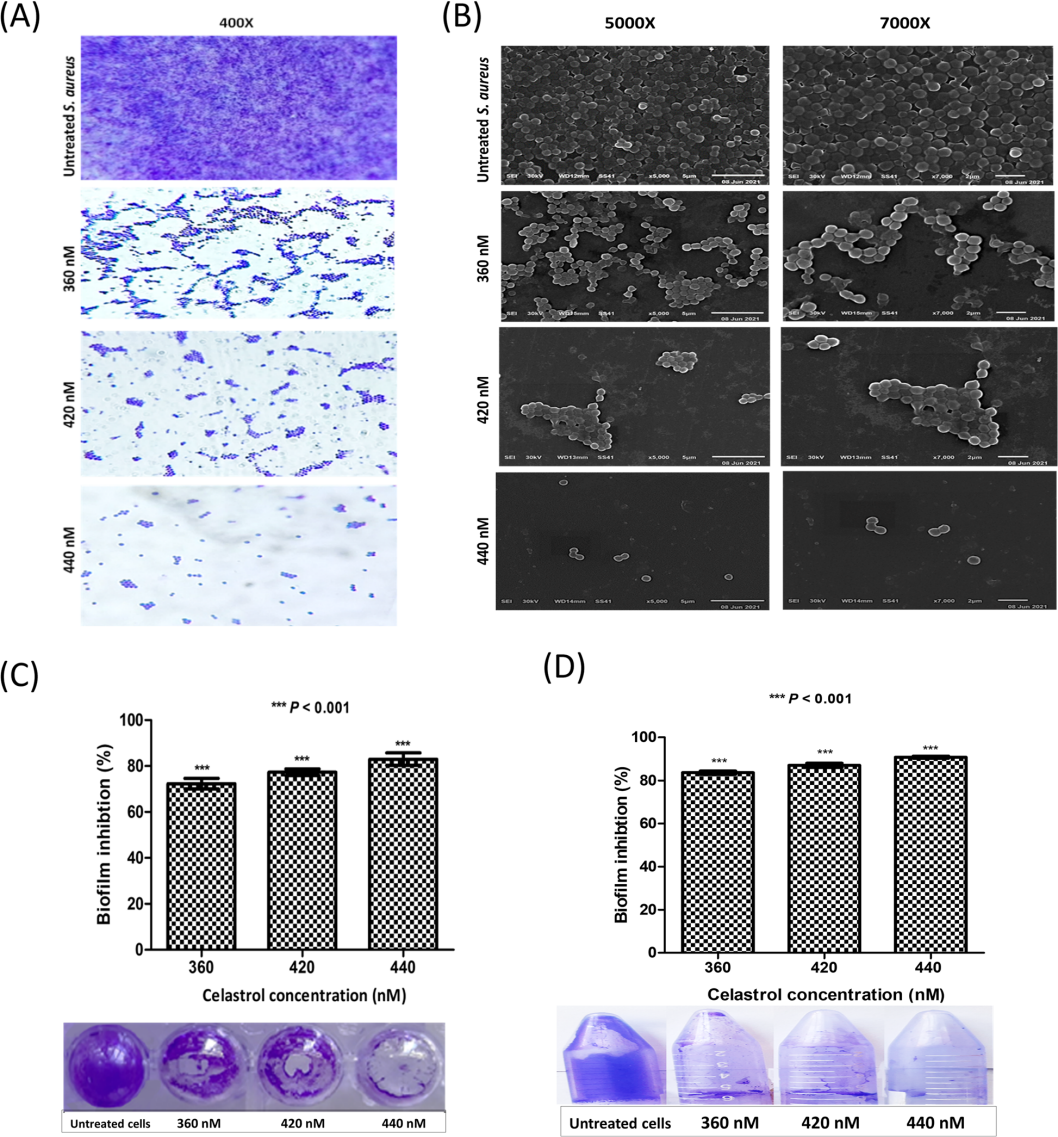
Concentration-dependent inhibitory activity of celastrol on biofilm formation as revealed by; (**A**) light microscopy and (**B**) SEM images. Percentage of biofilm inhibition by celastrol evaluated by the crystal violet quantification assay on; (**C**) polystyrene plate and (**D**) polypropylene tube. Data shown represent the mean ± standard of error from triplicate experiments. A *P* value < 0.05 was considered statistically significant using Student’s *t* test

Light and scanning electron microscopy (SEM) analysis were conducted to investigate the anti-biofilm potential of celastrol against *S. aureus* ATCC 6538 biofilms. Regarding light microscopy analysis, images revealed a well-arranged and condensly aggregated biofilm structure formed by *S. aureus* in the absence of celastrol. In contrast, *S. aureus* microcolony development and aggregation were obviously disrupted upon celastrol treatment (Fig. 6A). In context with light microscopy, SEM analysis indicates a reduced biofilm formation by celastrol treated *S. aureus* in contrast to the dense biofilm formed by untreated cells (Fig. 6B). Furthermore, the activity of celastrol at increasing concentrations (360, 420 and 440 nM) was evaluated against *S. aureus* biofilm formation on both polystyrene and polypropylene surfaces, which are commonly used in the manufacture of indwelling medical devices. Results indicated a dose-dependent anti-biofilm activity of celastrol on both tested surfaces (Fig. 6C-D).

### Celastrol affects the expression of genes involved in STX biosynthesis, biofilm formation and *S. aureus* virulence

**Fig. 7:**
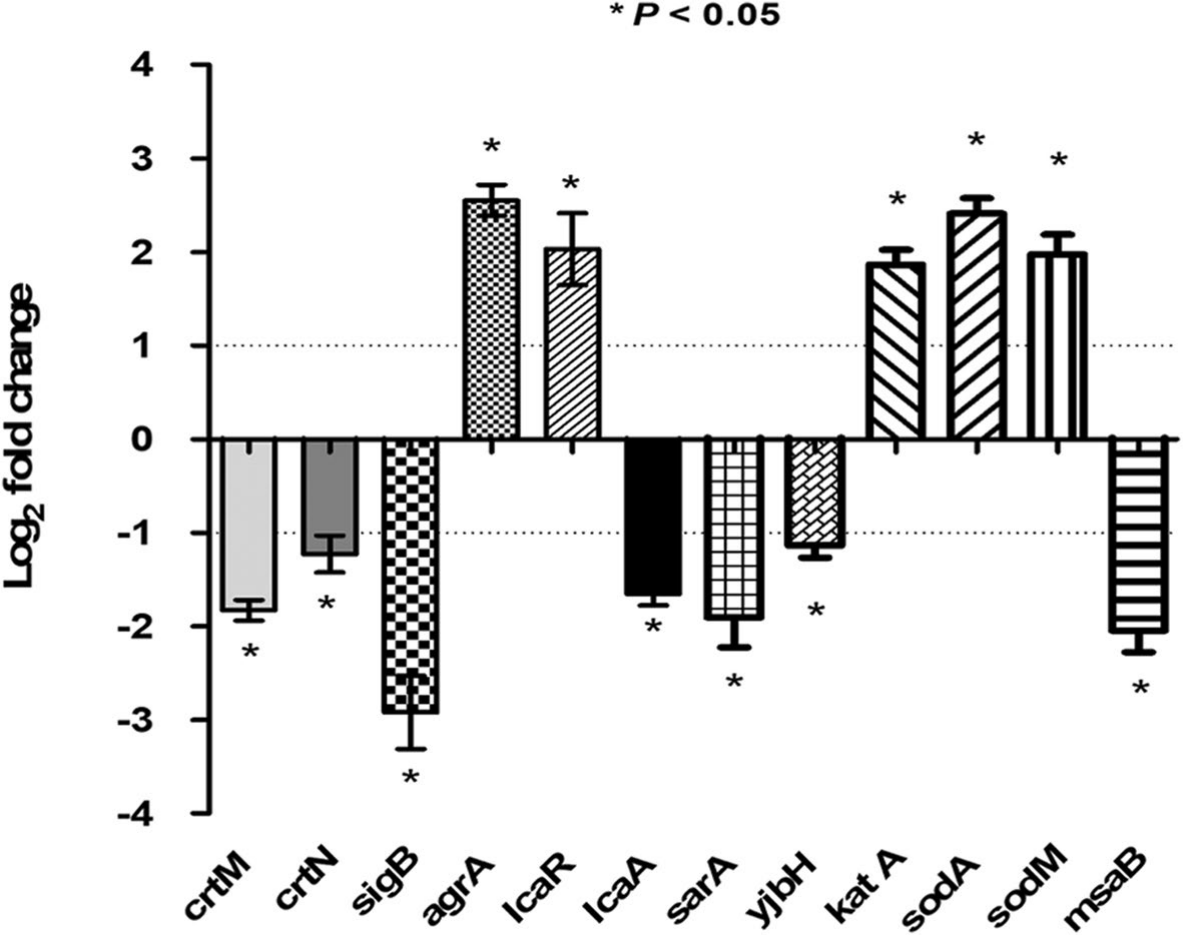
Celastrol altered the expression of *S. aureus* genes. RT-qPCR revealed decreased expression of STX and biofilm related genes in celastrol treated *S. aureus* relative to untreated bacteria. The expression levels of *crtM*, *crtN*, *sigB*, *katA*, *sodM*, *sodA*, *sarA*, *agrA, icaA*, *icaR*, *msaB* and *yjbH* were analyzed by qRT-PCR in both celastrol treated and untreated *S. aureus*. The data shown are the means ± standard errors from triplicate experiments with three technical replicates each. A *P* value < 0.05 was considered statistically significant using Student’s *t* test

RT-PCR analysis demonstrated that celastrol treatment significantly repressed the expression of STX biosynthesis and biofilm related genes such as *crtM, crtN, sarA,* and *icaA*. In addition, the expression of transcription regulators *msaB* and *sigB,* as well as stress adaptor *yjbH*, which play a major role in bacterial virulence, was found to be significantly downregulated in celastrol treated *S. aureus* relative to untreated cells. On the other hand, the oxidative stress responsive genes (*katA*, *sodA* and *sodM*) and the regulators *agrA* and *icaR* that are involved in the regulation of biofilm formation were shown to be upregulated (Fig. 7).

### Celastrol synergizes antibiotic inhibitory effect on *S. aureus*

The synergistic potential of celastrol with commonly used antibiotics like ampicillin, cefotaxime, azithromycin, ciprofloxacin and gentamicin to inhibit planktonic *S. aureus* ATCC 6538 was determined. Synergy was studied by the checkerboard method by determining the FICI (fractional inhibitory concentration index). Importantly, the MIC values for tested antibiotics were reduced when combined with celastrol, giving FIC values ≤ 0.5 indicating a synergistic effect (Table 2). The combination MICs of ampicillin, cefotaxime, azithromycin, ciprofloxacin, and gentamicin on *S. aureus* ATCC 6538 dropped to 0.5, 0.25, 0.06, 0.25 & 0.12 μg/mL in contrast to the individual MICs (8, 4, 1, 2 & 2 μg/mL), respectively. All tested antibiotics in combination with celastrol show a synergistic effect with FICIs ranging from 0.12 to 0.31.

**Table 2:** MICs and FICIs of celastrol and tested antibiotics against *S. aureus* ATCC 6538

Agent	MIC (μg∕mL)		FIC	FICI	Interpretation
	Alone	Combination			
**Celastrol**	**1**	**0.25**	**0.25**	**0.31**	**S**
**Ampicillin**	**8**	**0.5**	**0.06**		
**Celastrol**	**1**	**0.25**	**0.25**	**0.31**	**S**
**Cefotaxime**	**4**	**0.25**	**0.06**		
**Celastrol**	**1**	**0.06**	**0.06**	**0.12**	**S**
**Azithromycin**	**1**	**0.06**	**0.06**		
**Celastrol**	**1**	**0.06**	**0.06**	**0.185**	**S**
**Ciprofloxacin**	**2**	**0.25**	**0.125**		
**Celastrol**	**1**	**0.06**	**0.06**	**0.12**	**S**
**Gentamycin**	**2**	**0.12**	**0.06**		

***FIC*** Fractional inhibitory concentration, ***FICI*** Fractional inhibitory concentration index, ***S*** Synergism

### Celastrol reduces *S. aureus* pathogenesis in mice infection model

**Fig. 8:**
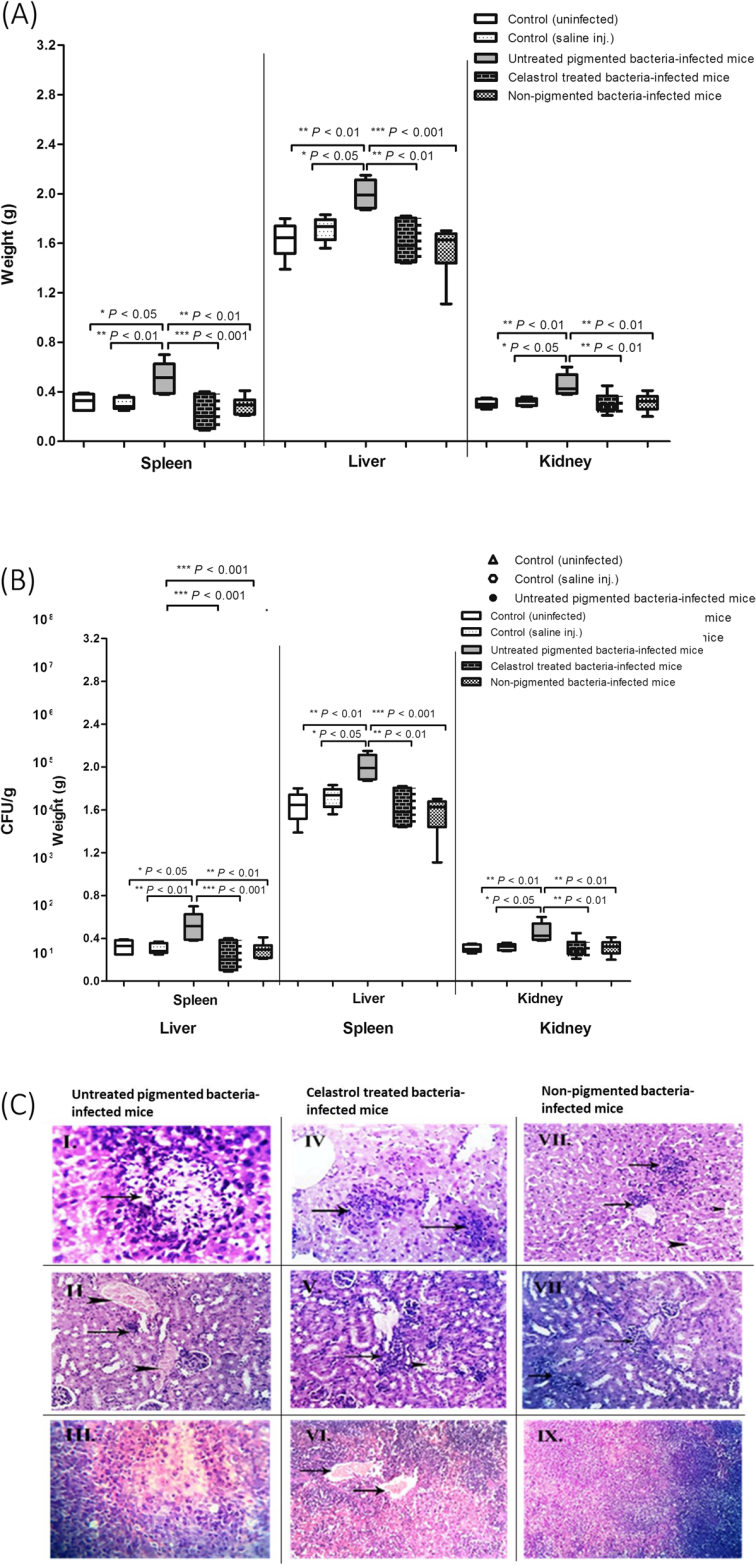
In vivo efficacy of celastrol against *S. aureus* infection. (**A**) Organ weight change of inoculated mice with significant increase in pigmented bacteria-infected mice. (**B**) Bacterial load of liver, spleen, and kidney of each group. (**C**) Histopathological organs section from pigmented, celastrol treated and non-pigmented bacteria-infected mouse stained by hematoxylin and eosin stain; (I) Liver focal necrotic area with leucocyte infiltration. (II) Kidney focal fibrosis (arrow) with severe congestion (arrowheads). (III) Spleen parenchyma with focal necrotic area. (IV) Liver focal leucocytes infiltration (arrows). (V) Kidney focal leucocytic infiltration (arrow) with degeneration of some renal tubules (arrowhead). (VI) Spleen blood vessels congestion (arrows). (VII) Liver focal perivascular infiltration within von Kupffer cells (arrows) and dilated sinusoids (arrowheads). (VIII) Kidney hypercellularity of few glomeruli. (IX) Spleen with normal white and red pulp. Each symbol represents the value for an individual mouse and horizontal bars indicate the means**.** A *P* value < 0.05 was considered statistically significant using Mann–Whitney *U* analysis

Organ weights were assessed for both mice infected with *S. aureus* ATCC 6538 (untreated and celastrol treated) and a non-pigmented *S. aureus* isolate. Mice organs infected with pigmented bacteria were heavier than those isolated from the negative control mice group mice injected with celastrol treated bacteria and non-pigmented isolate (Fig. 8A). In addition, the bacterial burden in mouse organs was assessed for both control and bacteria-inoculated mice. The results show that pigmented *S. aureus* colonized mice tissues significantly more than celastrol-treated bacteria (Fig. 8B). Furthermore, histopathological examination of organ tissues isolated from mice infected with pigmented *S. aureus* revealed abnormal tissue pathology with irreversible tissue necrosis and fibrosis. In contrast, organ tissues isolated from mice infected with celastrol treated and non-pigmented *S. aureus* showed immature lesions with mild inflammatory signs like blood vessel congestion and localized leucocyte infiltration (Fig. 8C).

## Discussion

*S. aureus* coordinates an array of virulence factors in order to wreak havoc on the body [43]. One of *S. aureus* characteristic virulence factors is STX, which desensitizes bacterial cells towards host immune clearance [7]. In addition, biofilm formation enhances *S. aureus* resistance to antibiotics and bacterial survival under various environments [9]. Therefore, the introduction of novel anti-virulent agents such as STX or biofilm inhibitors has become necessary to control *S. aureus* infectivity [44]. The present study demonstrates for the first time that celastrol could be a potential agent for the control of *S. aureus* infections. Current data clearly indicates that celastrol has significant anti-pigment and anti-biofilm activities against *S. aureus*.

Many factors could affect STX production by *S. aureus.* Staphylococcal STX production has been shown to be more pronounced in well-aerated conditions (Fig. S1A). Bacterial exposure to oxygen leads to redox-signaling of AirS [2Fe-2S]^2+^ cluster and transcriptional activation of AirR which activates transcription of *CrtOPQMN* with more STX production [45]. On the other hand, extended bacteria culturing time led to a lower abundance of carotenoids, which could be explained by the depletion of medium nutrients in the late stationary phase. Wieland et al. [46] found that there was more STX at 24 h of cell growth than at 36 h of cell growth, which could be related to variations in medium metabolites detected in both the exponential and stationary phases [47]. Regarding medium nutrient effect, *S. aureus* growth in TSB and MHB resulted in increased STX biosynthesis. It has been shown that *S. aureus* produces higher levels of branched chain fatty acids in these media, which leads to an increase in carotenoid production [48]. Furthermore, pigment biosynthesis was found to be enhanced upon glucose addition to culture media, since glucose is required for STX production [8, 49].

Importantly, the present study unveiled celastrol inhibitory potential on bacterial STX production. Celastrol inhibits pigment biosynthesis without affecting either bacterial cell viability or metabolic activity. Treatment of *S. aureus* with celastrol interferes with the biosynthesis of STX intermediates; 4,4′-diapophytoene, 4,4′-diaponeurosporene and 4,4′diaponeurosporenic acid. LC-MS analysis validated this observation and demonstrated the accumulation of farnesyl diphosphate, which is a substrate for the dehydrosqualene synthase CrtM. Furthermore, molecular docking analysis revealed a possible interaction between celastrol and staphylococcal CrtM. In accordance with current results, Song et al. [21] reported that celastrol is a non-competitive inhibitor of fungal squalene synthase, which shares a structural similarity with *S. aureus* CrtM. Moreover, qRT-PCR indicated that celastrol treated bacterial cells revealed a significant repression of *crtM* as well as the *crtOPQMN* operon regulators, *sigB*, *msaB* and *yjbH,* which further confirms current findings [36, 50, 51].

The present study demonstrates that celastrol shows an inhibitory effect on *S. aureus* intracellular survival as it sensitizes cells to oxidants and acidic pH. Being a carotenoid, STX works as an antioxidant, which could protect *S. aureus* against oxidative stress [7, 52]. Celastrol treated *S. aureus* was more sensitive to oxidative stress by H_2_O_2_ and human blood cells. Similarly, celastrol treated cells were less tolerant to acid, which would make cells more readily killed by the acidic pH of phagolysosomes [53]. In addition to inhibition of STX biosynthesis, the enhanced susceptibility of celastrol treated cells to both oxidative and acid stress could be attributed to the downregulation of *sig*B, *yjbH* and *msaB*, which have been shown to be important for *S. aureus* survival under stressful environments [36, 50, 51]. Accordingly, repression of *sigB* lead to increased expression of *katA*, *sodA* and *sodM,* which are known to be involved in Staphylococcal response to oxidative stress [54, 55].

The saccharolipid STX was found to have a significant effect on the fluidity of *S. aureus* membrane [56]. FTIR analysis of celastrol treated *S. aureus* showed spectral differences when compared with untreated cells, which represents changes in membrane lipid composition [57]. These changes in *S. aureus* cell membrane behaviour upon celastrol treatment could be due to the reduced STX content. Furthermore, owing to the fact that STX affects cell wall rigidity, *S. aureus* has an intrinsic resistance to membrane acting antibiotics, such as polymyxin B [48, 58]. Polymyxin B binds to cell wall lipopolysaccharides, and subsequently inserts itself into the cell membrane, which distorts membrane homeostasis. Current results indicate that celastrol significantly potentiates the inhibitory action of polymyxin B on *S. aureus.* Celastrol treated cells were more susceptible to polymyxin B relative to untreated cells. These findings further confirm the importance of STX for *S. aureus* resistance to antimicrobials and hence a potential role for celastrol in controlling infections by *S. aureus*.

Microbial biofilms cause serious health problems for patient requiring indwelling medical devices. Bacteria in biofilms are difficult to treat with antimicrobial agents [59]. Polystyrene and polypropylene plastic have been extensively used in the manufacture of indwelling medical devices and are prone to biofilm formation [60]. Current results show that celastrol significantly inhibited biofilm formation on both polypropylene and polystyrene surfaces. The anti-biofilm activity exhibited by celastrol could be explained by celastrol-induced repression of the *S. aureus* biofilm regulators *sarA* and *msaB*. It has previously been demonstrated that *sarA* and *msaB* mutations reduce *S. aureus* ability to form a biofilm [61]. Furthermore, it has been shown that *S. aureus agr* quorum sensing system controls the switch between planktonic and biofilm lifestyles. The *agr* system impedes the biofilm development via suppressing the production of adhesion proteins and inducing the expression of matrix degrading enzymes such as protease and nuclease [62]. In line with Shukla and Rao [63], our findings suggest that *agrA* upregulation activated quorum sensing system, which in turn induced the expression of degrading enzymes and biofilm dispersal.

*S. aureus* produces a polysaccharide intercellular adhesin (PIA) encoded by *icaADBC* operon. PIA is responsible for mediating cell-cell adhesion and protecting bacteria from both host immune defenses and antimicrobial agents [11]. Importantly, celastrol shows an inhibitory effect on EPS production and consequently, on *S. aureus* biofilm formation (Fig. S4A). Bacterial treatment with celastrol resulted in the downregulation of *icaA* as well as *sarA* and *sigB* that positively regulate *icaADBC* operon. Furthermore, *icaR* was upregulated upon celastrol treatment, which is a negative regulator of *icaADBC* operon [64]. Bacterial cell auto-aggregation and surface hydrophobicity have been shown to markedly contribute to biofilm formation. For instance, bacterial biofilm formation is greatly enhanced by cell auto-aggregation, which also influences bacterial pathogenesis through promoting invasion of host cells [65] and increasing survival within phagosomes [66]. The present study indicates that *S. aureus* auto-aggregation was significantly interrupted upon treatment with celastrol (Fig. S4B). Similarly, bacterial surface hydrophobicity plays an important role in biofilm formation [65]. Celastrol revealed a potent inhibition of *S. aureus* surface hydrophobicity (Fig. S4C). Celastrol could trigger *S. aureus* surface hydrophilicity and thereby reduce cell hydrophobicity, which interferes with bacterial attachment to hydrophobic surfaces [67]. All these findings clearly suggest that celastrol is a promising anti-biofilm and anti-pathogenic agent.

It has been shown that phytochemicals like celastrol could be used as antibacterials or in combination with antibiotics as adjuvants [68]. The present study assessed the synergistic potential of celastrol with conventional antibiotics. In line with that, celastrol treated cells were highly susceptible to induced autolysis relative to untreated cells (Fig. S5). Celastrol increased autolytic activity was attributed to down regulation of *msaB*, which has been reported to regulate the rate of cell death [69]. This increased bacterial autolytic activity significantly synergizes the bactericidal activity of cell wall-active antibiotics [70]. Celastrol in combination with ampicillin and cefotaxime resulted in a 16-fold reduction in the MIC values of tested antibiotics. In addition to increased autolysis upon treatment with celastrol, this high magnitude of reduction in antibiotic resistance may be due to functional membrane microdomain (FMM) disassembly induced by STX inhibition. It has been indicated that perturbation of FMM assembly could interfere with PBP2a oligomerization, rendering *S. aureus* more susceptible to penicillin treatment [71, 72]. Furthermore, decreased msaB expression in *S. aureus* after celastrol treatment may result in decreased peptidoglycan cross-linking and increased susceptibility to cell wall-targeting antibiotics such as ß-lactams [69]. Similarly, celastrol potentiates the activities of azithromycin, ciprofloxacin and gentamycin against *S. aureus*. Azithromycin has been shown to be an effective therapeutic option against MRSA infections associated with α-hemolysin production and biofilm formation [73]. On the other hand, ciprofloxacin and gentamycin cause altered cellular respiration and induce lethal levels of intracellular damaging reactive species [74]. In order to establish pathogenesis, *S. aureus* has to overcome oxidative stress exerted by aerobic respiration and host pathogen interaction [75]. Celastrol as a STX inhibitor could sensitize *S. aureus* to oxidative stress. Combination of celastrol with ciprofloxacin or gentamicin could disrupt bacterial tolerance to oxidative stress, which represents a potential synergistic approach to combat *S. aureus*. Overall, these findings indicate that celastrol is an efficient compound to offset antibiotic resistance.

Finally, the importance of STX for *S. aureus* pathogenesis was characterized in vivo using a murine infection model. Bacterial colonization of mouse organs infected with celastrol treated *S. aureus* was compared with the control mice injected with untreated pigmented and non-pigmented bacteria. Interestingly, organ weight increased significantly after bacterial challenge with untreated pigmented *S. aureus* in comparison to control uninfected mice, which could be attributed to inflammtory response accompanied with bacterial colonization. These findings are completely in agreement with previous studies [76, 77] that reported that mice inoculation with virulent bacteria results in increased organ weight relative to control uninfected mice. Both celastrol treated and non-pigmented *S. aureus* were less virulent in mice and thus less able to colonize mouse organs than untreated pigmented bacteria. Moreover, the lesions generated by celastrol treated and non-pigmented bacteria were milder and more immature than those generated by untreated pigmented bacteria. As mentioned above, celastrol treated cells are defective in both STX biosynthesis and biofilm formation, which could account for the decreased bacterial virulence in mice. In addition, the decreased pathogenesis of *S. aureus* upon treatment with celastrol could be further explained by the downregulation of many genes involved in bacterial virulence, such as *sarA* and *yjbH*. Disruption of *sarA* loci, for example, has been shown to reduce virulence in several infection models [78, 79]. Furthermore, Paudel et al. [51] reported that *yjbH* is essential for *S. aureus* full virulence where it regulates many surface proteins and virulence genes and is important for oxidative stress survival within the host.

## Conclusion

Collectively, the current study unveils the importance of celastrol as an anti-virulence agent against *S. aureus*. Celastrol exhibits anti-staphyloxanthin activity, sensitising *S. aureus* to hydrogen peroxide as well as human blood killing. *S. aureus* cell membrane rigidity significantly decreases following celastrol treatment, rendering bacteria more susceptible to membrane-targeting antibiotics. In addition, celastrol possesses a higher anti-biofilm activity along with its inhibitory effect on bacterial cell EPS, hydrophobicity and auto-aggregation. This anti-biofilm activity is owed to the downregulation of biofilm related genes such as *icaA* and global regulators *sarA* and *msaB*. Interestingly, celastrol markedly attenuated *S. aureus* pathogenesis in mice infection model*.* The net outcome of celastrol effects on *S. aureus* virulence could be a complex of different activities. Furthermore, this study demonstrates that celastrol could modulate the activity of many global regulators such as SigB, SarA and MsaB as well as the stress adaptor *yjbH* that play a pronounced role in bacterial survival and pathogenesis. With the envisioning of a future practical application of present findings, celastrol could play a role in the management of staphylococcal infections.

## Supplementary Information

**Additional file 1: Data S1** Additional methods**. Fig*.*** **S1** Conditions affecting STX production. **Fig. S2** Celastrol did not interfere with *S. aureus* viability or metaboilic activity at sub-MIC. **Fig. S3** In vitro pigment inhibition of clinical isolates by celastrol. **Fig. S4** Dose dependent inhibition of EPS production, cell auto-aggregation and hydrophobicity index of *S. aureus* cells upon celastrol treatment. **Fig. S5** Celastrol increased Triton X-100 induced autolytic activity of *S. aureus****.*** **Fig. S6** Celastrol had no effect on esterase, lipase and DNase enzyme production.

## Data Availability

The authors confirm that the data supporting the findings of this study are available within the article.

## Abbreviations

LC-MS: Liquid chromatography-mass spectrometry
STX: Staphyloxanthin
ROSs: Reactive oxygen species
PIA: Polysaccharide intracellular adhesion
TSB: Trypticase soy broth
CFUs: Colony forming units
PBS: Phosphate buffer saline
MIC: Minimum inhibitory concentration
MHB: Mueller Hinton broth
IC_50_: Half maximal inhibitory concentration
PDB: Protein databank
TSA: Tryptone soya agar
FTIR: Fourier-transform infrared spectroscopy
CV: Crystal violet
qRT-PCR: Quantitative real time PCR
AMP: Ampicillin
CTX: Cefotaxime
CIP: Ciprofloxacin
AZM: Azithromycin
CN: Gentamycin
FICI: Fractional inhibitory concentration index
FIC: Fractional inhibitory concentration
SEM: Scanning electron microscope
FMM: Functional membrane microdomain
