# Correction: Celastrol mitigates staphyloxanthin biosynthesis and biofilm formation in Staphylococcus aureus via targeting key regulators of virulence; in vitro and in vivo approach

**DOI:** 10.1186/s12866-022-02538-6

**Published:** 2022-05-13

**Authors:** Fatma Al-zahraa A. Yehia, Nehal Yousef, Momen Askoura

**Affiliations:** Department of Microbiology and Immunology, Faculty of Pharmacy, Zagazig University, Zagazig, 44519 Egypt

**Correction to: BMC Microbiol 22(1):106 (2022)**

**https://doi.org/10.1186/s12866-022-02515-z**

Following the publication of the original article [1], the authors spotted error on figure. Figure 8a was repeated and superimposed on Figure 8b which means that Figure 8b has been deleted from the paper. The missing image is shown below.

**Fig. 8:**
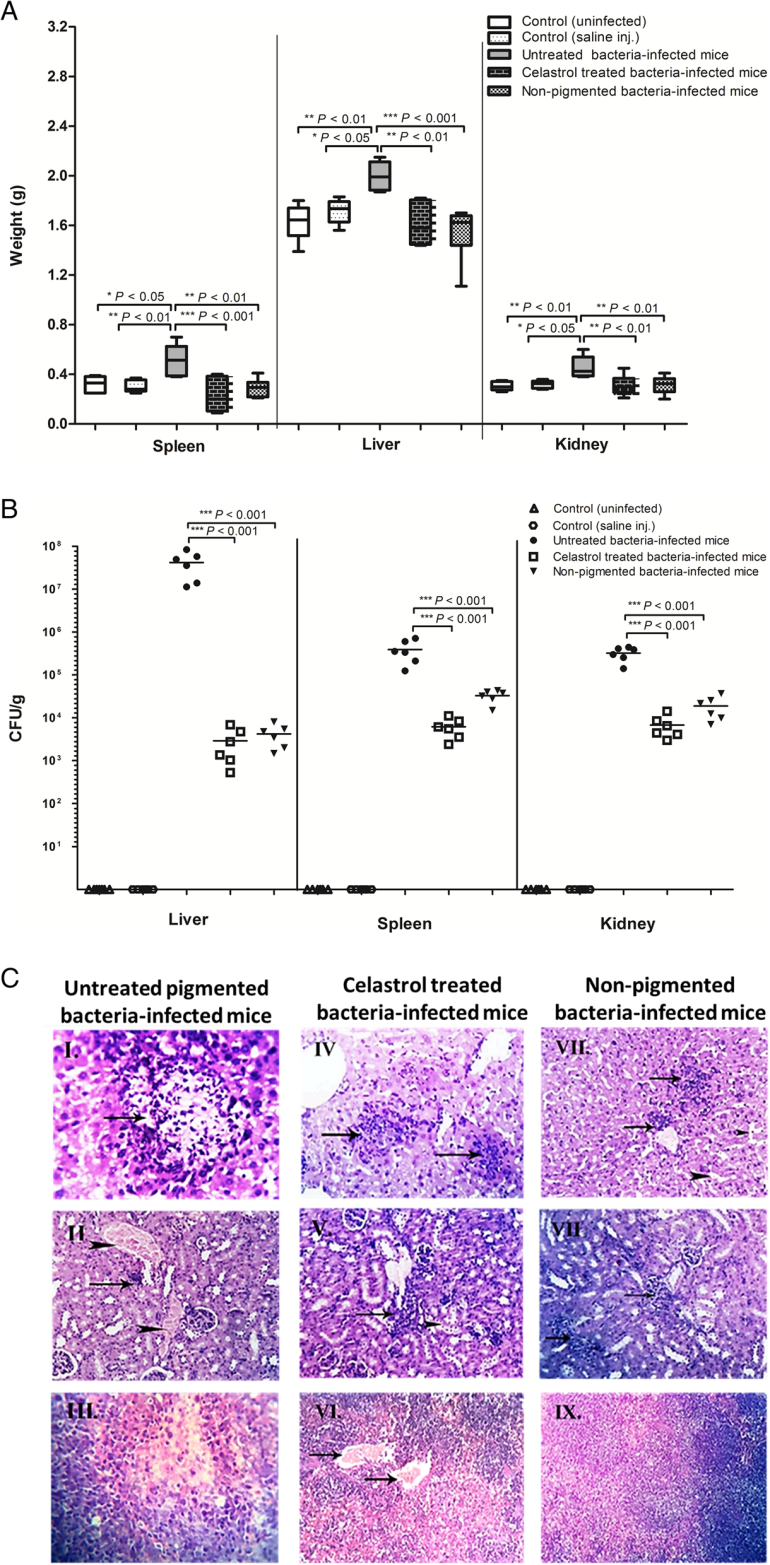
In vivo efficacy of celastrol against *S. aureus* infection. (**A**) Organ weight change of inoculated mice with significant increase in pigmented bacteria-infected mice. (**B**) Bacterial load of liver, spleen, and kidney of each group. (**C**) Histopathological organs section from pigmented, celastrol treated and non-pigmented bacteria-infected mouse stained by hematoxylin and eosin stain; (I) Liver focal necrotic area with leucocyte infiltration. (II) Kidney focal fibrosis (arrow) with severe congestion (arrowheads). (III) Spleen parenchyma with focal necrotic area. (IV) Liver focal leucocytes infiltration (arrows). (V) Kidney focal leucocytic infiltration (arrow) with degeneration of some renal tubules (arrowhead). (VI) Spleen blood vessels congestion (arrows). (VII) Liver focal perivascular infiltration within von Kupffer cells (arrows) and dilated sinusoids (arrowheads). (VIII) Kidney hypercellularity of few glomeruli. (IX) Spleen with normal white and red pulp. Each symbol represents the value for an individual mouse and horizontal bars indicate the means. A *P* value < 0.05 was considered statistically significant using Mann–Whitney U analysis
