# Supplementary material for: Celastrol mitigates staphyloxanthin biosynthesis and biofilm formation in Staphylococcus aureus via targeting key regulators of virulence; in vitro and in vivo approach
Source: BMC Microbiol. 2022 Apr 15;22:106. doi: 10.1186/s12866-022-02515-z (PMC9011992; doi:10.1186/s12866-022-02515-z)
Supplement: Supplementary file 1 — Additional file 1: Data S1 Additional methods. Fig. S1 Conditions affecting STX production. Fig. S2 Celastrol did not interfere with S. aureus viability or metaboilic activity at sub-MIC. Fig. S3 In vitro pigment inhibition of clinical isolates by celastrol. Fig. S4 Dose dependent inhibition of EPS production, cell auto-aggregation and hydrophobicity index of S. aureus cells upon celastrol treatment. Fig. S5 Celastrol increased Triton X-100 induced autolytic activity of S. aureus. Fig. S6 Celastrol had no effect on esterase, lipase and DNase enzyme production. [file 12866_2022_2515_MOESM1_ESM.docx]

# Supplementary methods

**Characterization of factors affecting staphyloxanthin production by *S. aureus***

The production of staphyloxanthin by *S. aureus* ATCC 6538 was investigated under various environmental conditions. These conditions include media nutrients, growth incubation time, culture aeration and light as follow. Effect of Nutrients: *S. aureus* was grown in different laboratory media (tryptone soya broth (TSB), Mueller Hinton broth (MHB), Luria-Bertani broth (LB), nutrient broth (NB) and nutrient broth supplemented with 0.5% glucose (NBG)). Effect of growth incubation time: bacterial cells were grown for different incubation time (24 h, 48 h and 72 h). Effect of culture aeration: cells were grown in various flask-to-medium ratios (vol/vol) of (50:2.5, 50:3, 50:5, 50:10 and 50:25). Photobleaching in ambient light: cells were grown for 24 h and the extracted carotenoids were subjected to ambient light for 1 h. For all conditions, carotenoid extraction and estimation was carried as described by (Kossakowska-Zwierucho, Kaźmierkiewicz, Bielawski, & Nakonieczna, 2016).

**Alamar blue assay**

To assess the effect of subminimum inhibitory concentration (sub-MIC) of celastrol on *S. aureus* metabolic activity, the alamar blue assay was conducted as described by Gargotti *et al.* (Gargotti, Lopez-Gonzalez, Byrne, & Casey, 2018)*.* Briefly, *S. aureus* was grown in TSB with celastrol (360, 420 and 440 nM). After incubation, cell pellets were harvested by centrifugation at 8000 rpm and resuspended in PBS. Resazurin (Sigma-Aldrich) stock solution was prepared as 6.5 mg/mL in PBS. Bacterial suspension (0.9 mL) and resazurin solution (0.1 mL) were added and incubated at 37°C in the dark for 4 h. The intensity of the supernatant fluorescence was observed at wavelengths (590 nm for emission and 560 nm for excitation) and compared with untreated bacteria and PBS containing resazurin as blank.

**Exopolysaccharide (EPS) assay**

The phenol-sulfuric acid method was carried for the total quantification of EPS released in untreated and celastrol treated *S. aureus* (Favre-Bonté, Köhler, & Van Delden, 2003). Briefly, sterile glass slides were immersed in TSBG containing *S. aureus* with celastrol (360, 420 and 440 nM). After incubation, the glass slides were removed and washed. Bacterial suspension in PBS was collected, mixed with phenol (5%) and H_2_SO_4_ and incubated for 1 h in dark. The supernatant absorbance was measured at 490 nm and compared with untreated bacteria.

**Auto-aggregation assay**

The effect of celastrol on bacterial cell auto-aggregation was determined (Sorroche, Spesia, Zorreguieta, & Giordano, 2012). Briefly, *S. aureus* ATCC 6538 was incubated in TSBG with various concentrations of celastrol (360, 420 and 440 nM). Bacterial cells were centrifuged at 12,000 rpm and washed. Cell pellet was resuspended in PBS and allowed to stand at room temperature for 20 h. Optical cell density of the upper portion of PBS containing cells was measured after 20 h at 600 nm and compared with untreated bacteria. The auto-aggregation was derived from change in turbidity of upper portion.

**Characterization of bacterial surface hydrophobicity index**

*S. aureus* surface hydrophobicity was evaluated as previously described (Nostro et al., 2004). *S. aureus* ATCC 6538 was incubated TSBG containing celastrol (360, 420 and 440 nM). Cells were harvested by centrifugation at 13000 rpm. The initial optical density (Ai) was adjusted to 1.0 ± 0.01 at a wavelength of 595 nm using a spectrophotometer. 200 µL of toluene was mixed with adjusted bacterial suspension (1 mL) and vortexed. After phase separation, the aqueous phase absorbance (Af) was secondly measured. The hydrophobicity index (HI) was expressed as: HI = (Ai − Af)/Ai ×100% and compared with HI of bacteria grown in absence of celastrol.

**Triton X-100 induced autolysis.**

Autolysis assay was carried by using Triton X- 100 as described by (Sahukhal, Batte, & Elasri, 2015).  *S. aureus* cultures were grown in TSB broth contain 1M NaCl in absence and presence of celastrol (440nM). Bacteria were incubated until the optical density reaches to 0.7 at 580 nm, the cells were harvested by centrifuging at 10,000 rpm for 10 min and washed twice. Finally, the cells were resuspended in autolysis buffer (50 mM Tris-HCl (pH 7.5) containing 0.1% (v/v) Triton X-100) and incubated with shaking. The rate of bacterial cells autolysis was monitored by measuring OD at 580 nm every 30 min intervals for 3 hrs. The decrease of optical density evidences the increased autolysis.

**Esterase, lipase and DNase qualitative assessment**

Tween [substrate plates](https://www.sciencedirect.com/topics/engineering/substrate-plate) (per liter H_2_O; 10 g peptone, 5 g NaCl_2_, 0.1 g CaCl_2_·2H_2_O, 20 g agar and 10 mL (v/v) Tween 20/80), as well as DNase plates were prepared. After solidification, wells were cut into agar medium and supernatants of untreated and celastrol treated *S. aureus* ATCC 6538 (440 nM) were added into wells. Esterase/lipase activity was observed after incubation at 37 °C for 2-4 days as white precipitation around the boundary of wells. However, DNase activity was observed after 24 h as a clear zone around well upon addition of 1N HCL (Jeffries, Holtman, & Guse, 1957; Plou et al., 1998).

**Supplementary results**


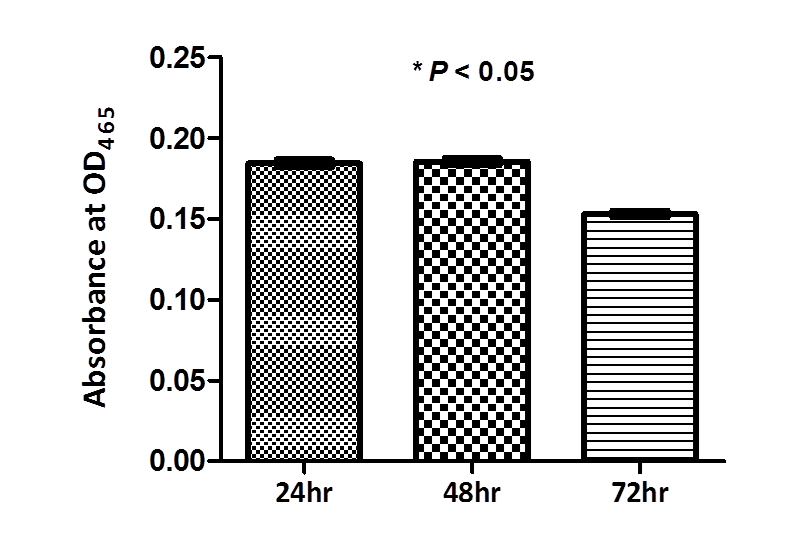


(c)


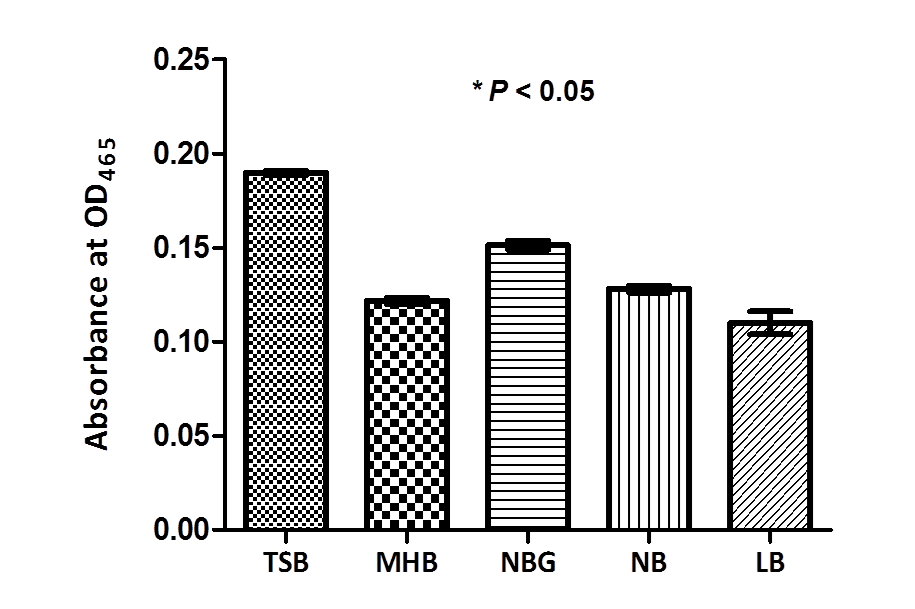


(a)


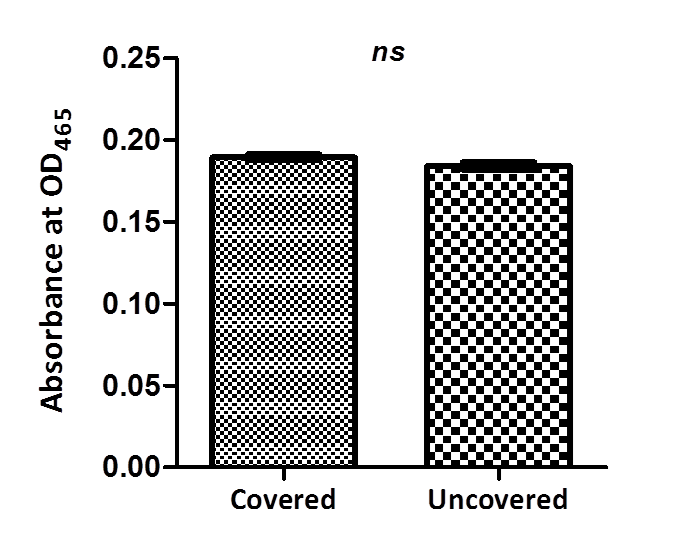


(d)


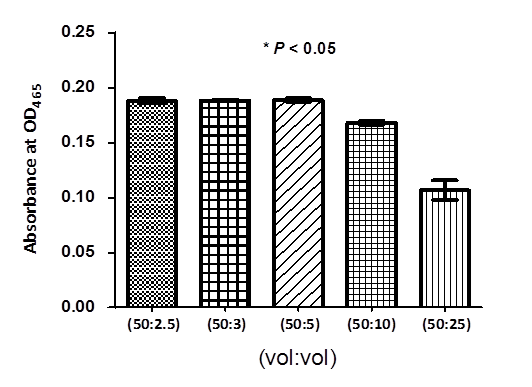


(b)

**Figure S1 Conditions affecting staphyloxanthin production;** (a) aeration, (b) incubation time, (c) media composition using laboratory media [tryptone soya broth (TSB), Mueller Hinton broth (MHB), Luria-Bertani broth (LB), nutrient broth (NB) and nutrient broth supplemented with 0.5% glucose (NBG)], (d) photobleaching at ambient light for one hour. Data shown represent the mean ± standard error from triplicate experiments. A *P* value < 0.05 was considered statistically significant using ANOVA test.


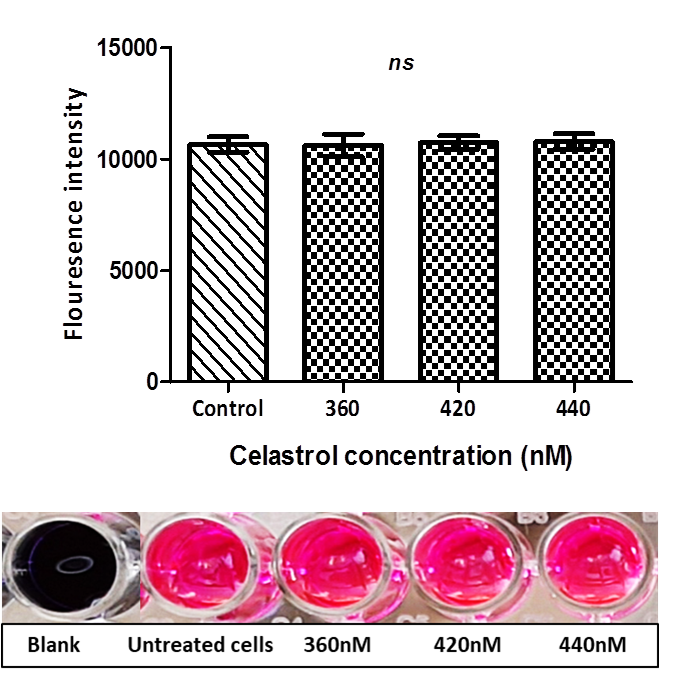


(b)

(a)


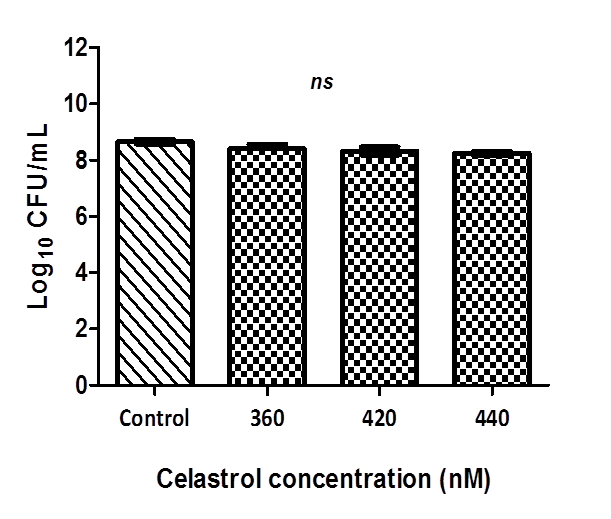


**Figure S2 Celastrol did not interfere with *S. aureus* viability or metaboilic activity at sub-MIC (360, 420 and 440 nM).** (a) CFU/mL of celastrol treated and untreated cells with no significant difference confirming non-bactericidal activity of sub-MIC (360, 420 and 440 nm). (b) Alamar blue assay confirming that celastrol treated cells were metabolically active as untreated cells. Data shown represent the mean ± standard error from triplicate experiments. (*ns* indicates no significant difference using Student’s *t* test.


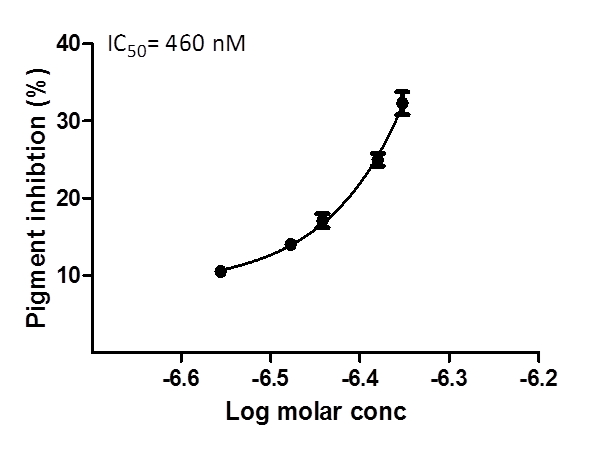


(c)


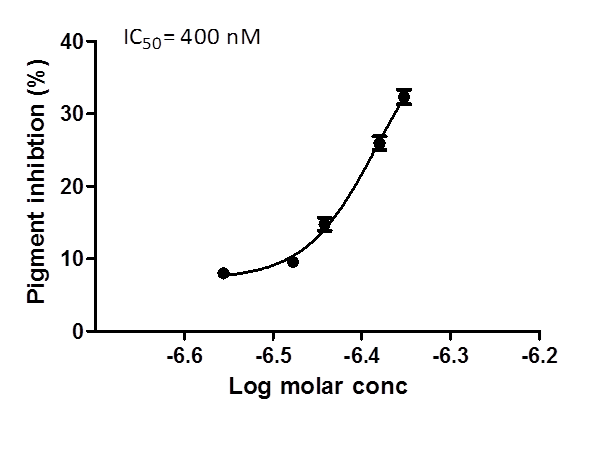


(a)

(b)

**
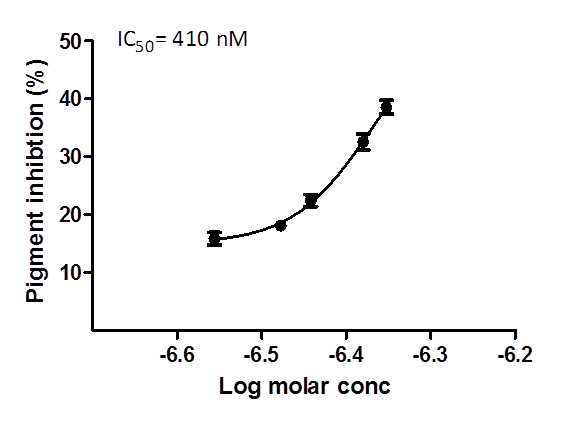
**


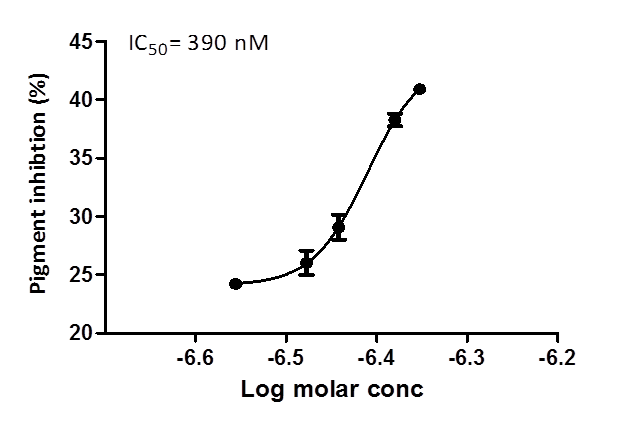


(b)

(d)

**Figure S3 *In vitro* pigment inhibition by celastrol.** Half maximal inhibitory concentrations (IC_50_) of celastrol against pigmented *S. aureus* clinical isolates from respiratory tract infection (a), burn infection (b), wound infection (c) and urinary tract infection (d). Data shown represent the mean ± standard error from triplicate experiments.


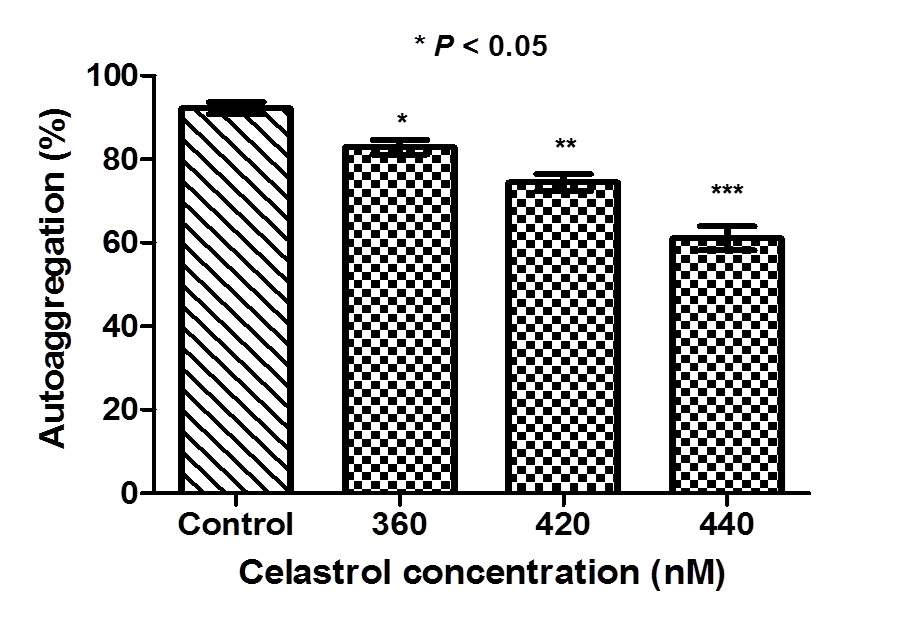


(b)


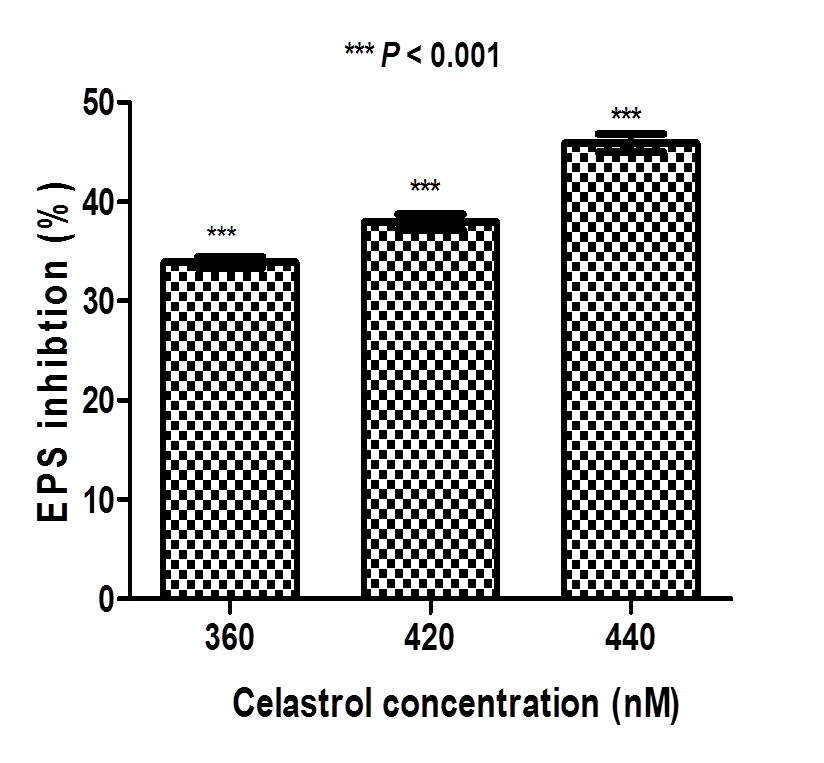


(a)


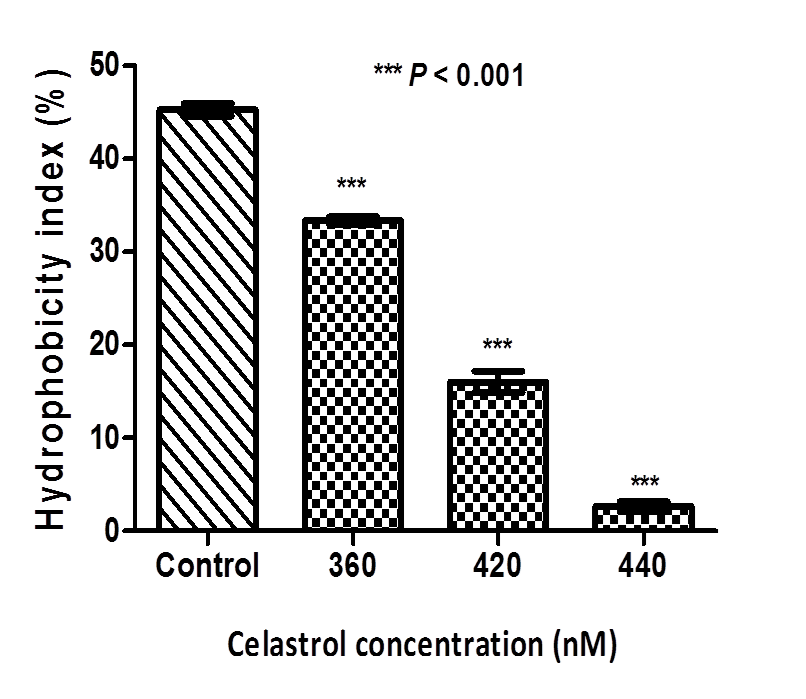


(c)


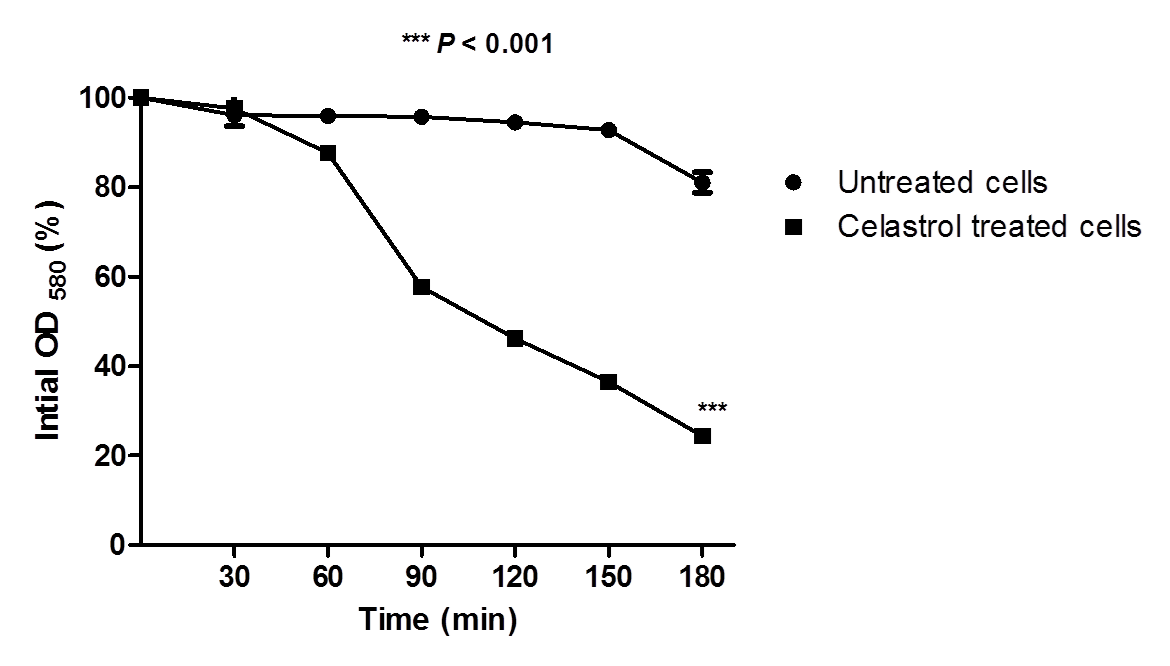
**Figure S4** (a) Dose dependent EPS inhibition in celastrol treated *S. aureus*. (b) Dose dependent reduction in auto-aggregation of *S. aureus* ATCC 6538 upon treatment with celastrol. (c) Dose dependent reduction of hydrophobicity index in celastrol treated *S. aureus* as compared to untreated control cells. Data shown represent the mean ± standard of error from triplicate experiments. A *P* value < 0.05 was considered statistically significant using Student’s *t* test.

**Figure S5 Celastrol increased Triton X-100 induced autolytic activity in *S. aureus.*** Data shown represent the mean ± standard of error from triplicate experiments. A *P* value < 0.05 was considered statistically significant using Student’s *t* test.

**
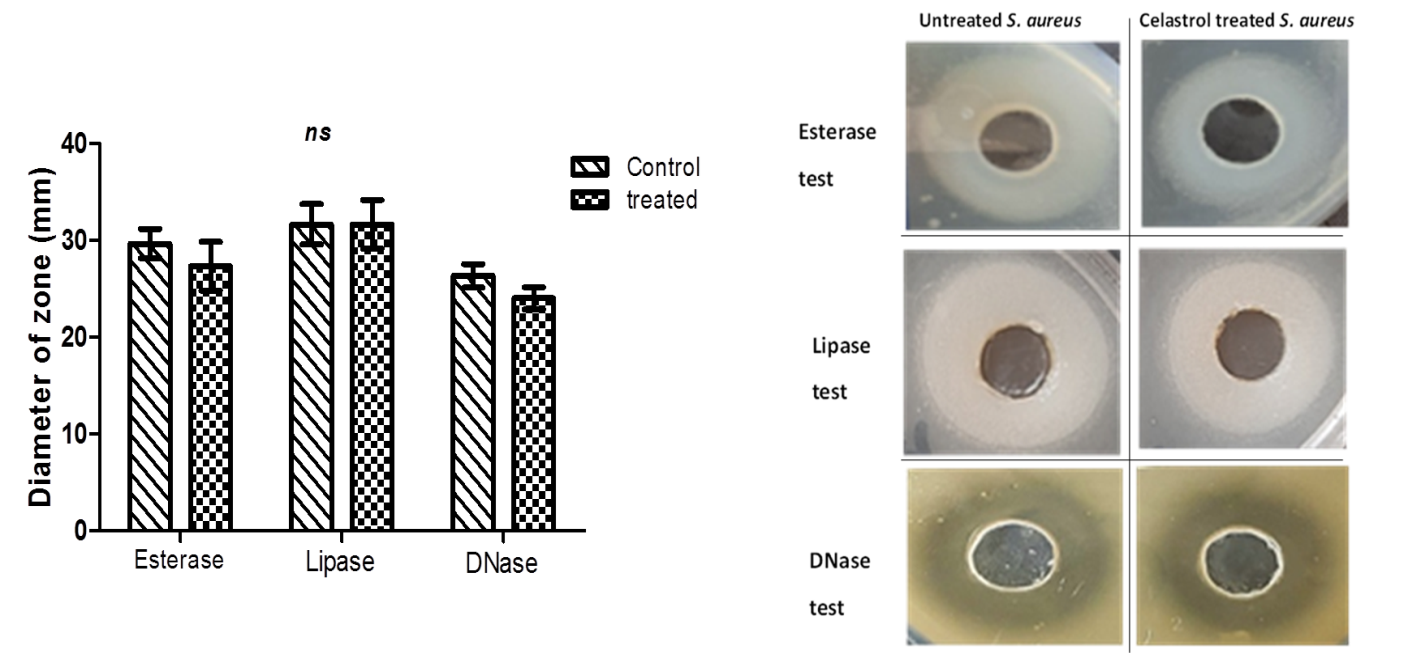
**

**Figure S6 Celastrol had no effect on esterase, lipase and DNase enzyme production.**  Data shown represent the mean ± standard of error from triplicate experiments. (*ns* indicates no significant difference using Student’s *t* test).

**Reference**

Favre-Bonté, S., Köhler, T., & Van Delden, C. (2003). Biofilm formation by Pseudomonas aeruginosa: role of the C4-HSL cell-to-cell signal and inhibition by azithromycin. *Journal of antimicrobial chemotherapy, 52*(4), 598-604. doi:10.1093/jac/dkg397

Jeffries, C. D., Holtman, D. F., & Guse, D. G. (1957). Rapid method for determining the activity of microorganisms on nucleic acids. *Journal of bacteriology, 73*(4), 590-591. doi:10.1128/JB.73.4.590-591.1957

Kossakowska-Zwierucho, M., Kaźmierkiewicz, R., Bielawski, K. P., & Nakonieczna, J. (2016). Factors Determining Staphylococcus aureus Susceptibility to Photoantimicrobial Chemotherapy: RsbU Activity, Staphyloxanthin Level, and Membrane Fluidity. *Frontiers in microbiology, 7*, 1141-1141. doi:10.3389/fmicb.2016.01141

Nostro, A., Cannatelli, M., Crisafi, G., Musolino, A., Procopio, F., & Alonzo, V. (2004). Modifications of hydrophobicity, in vitro adherence and cellular aggregation of Streptococcus mutans by Helichrysum italicum extract. *Letters in applied microbiology, 38*(5), 423-427.

Plou, F., Ferrer, M., Nuero, O., Calvo, M. V., Alcalde, M., Reyes, F., & Ballesteros, A. (1998). Analysis of Tween 80 as an esterase/ lipase substrate for lipolytic activity assay. *Biotechnology Techniques, 12*, 183-186. doi:10.1023/A:1008809105270

Sahukhal, G. S., Batte, J. L., & Elasri, M. O. (2015). msaABCR operon positively regulates biofilm development by repressing proteases and autolysis in Staphylococcus aureus. *FEMS microbiology letters, 362*(4), 1-10. doi:10.1093/femsle/fnv006

Sorroche, F. G., Spesia, M. B., Zorreguieta, A., & Giordano, W. (2012). A positive correlation between bacterial autoaggregation and biofilm formation in native Sinorhizobium meliloti isolates from Argentina. *Applied and environmental microbiology, 78*(12), 4092-4101. doi:10.1128/AEM.07826-11
